# Characteristics of glucose and lipid metabolism and the interaction between gut microbiota and colonic mucosal immunity in pigs during cold exposure

**DOI:** 10.1186/s40104-023-00886-5

**Published:** 2023-07-04

**Authors:** Teng Teng, Guodong Sun, Hongwei Ding, Xin Song, Guangdong Bai, Baoming Shi, Tingting Shang

**Affiliations:** College of Animal Science and Technology, Northeast Agricultural University, Harbin, 150030 China

**Keywords:** Cold exposure, Colonic mucosal immunity, Fatty acid oxidation, Glucose and lipid metabolism, Gut microbiota, Pig model

## Abstract

**Background:**

Cold regions have long autumn and winter seasons and low ambient temperatures. When pigs are unable to adjust to the cold, oxidative damage and inflammation may develop. However, the differences between cold and non-cold adaptation regarding glucose and lipid metabolism, gut microbiota and colonic mucosal immunological features in pigs are unknown. This study revealed the glucose and lipid metabolic responses and the dual role of gut microbiota in pigs during cold and non-cold adaptation. Moreover, the regulatory effects of dietary glucose supplements on glucose and lipid metabolism and the colonic mucosal barrier were evaluated in cold-exposed pigs.

**Results:**

Cold and non-cold-adapted models were established by Min and Yorkshire pigs. Our results exhibited that cold exposure induced glucose overconsumption in non-cold-adapted pig models (Yorkshire pigs), decreasing plasma glucose concentrations. In this case, cold exposure enhanced the ATGL and CPT-1α expression to promote liver lipolysis and fatty acid oxidation. Meanwhile, the two probiotics (*Collinsella* and *Bifidobacterium*) depletion and the enrichment of two pathogens (*Sutterella* and *Escherichia-Shigella*) in colonic microbiota are not conducive to colonic mucosal immunity. However, glucagon-mediated hepatic glycogenolysis in cold-adapted pig models (Min pigs) maintained the stability of glucose homeostasis during cold exposure. It contributed to the gut microbiota (including the enrichment of the *Rikenellaceae RC9 gut group*, *[Eubacterium] coprostanoligenes group* and *WCHB1-41*) that favored cold-adapted metabolism.

**Conclusions:**

The results of both models indicate that the gut microbiota during cold adaptation contributes to the protection of the colonic mucosa. During non-cold adaptation, cold-induced glucose overconsumption promotes thermogenesis through lipolysis, but interferes with the gut microbiome and colonic mucosal immunity. Furthermore, glucagon-mediated hepatic glycogenolysis contributes to glucose homeostasis during cold exposure.

**Supplementary Information:**

The online version contains supplementary material available at 10.1186/s40104-023-00886-5.

## Introduction

Cold poses a serious threat and is a major challenge for animals living at high latitudes. Climate phenomena like El Nino and La Nina, and global energy constraints, are undoubtedly making life worse for animals in cold regions. Numerous reports have examined the adverse consequences of cold stress on immunity, oxidation resistance, and animal growth [1–4]. When mammals are unable to adjust to low temperatures, excessive reactive oxygen species (ROS) production generation produces oxidative stress damage and mitochondrial malfunction, accompanied by inflammation [5–7]. Mammals experience a general alarm reaction, resistance and exhaustion in response to stressors [8]. Piglets are vulnerable to cold stress, which leads to colds, diarrhea, and even death, due to their lower immunity [9, 10]. Fast-growing pigs also tolerate cold exposure hardly. Our previous findings suggest that cold exposure induces intestinal mucosal dysfunction and causes lung injury in growing pigs, that are not accustomed to the cold [6, 11]. These undesirable phenomena undoubtedly increase the difficulty and cost of farms in cold regions during winter.

Reports from rodent models indicate that animals actively adjusting to the cold consume considerable energy substrates (glucose and triglycerides) [12, 13]. Glucose metabolism is one of the metabolic fluxes that coordinate energy homeostasis. The catabolism of glucose provides acetyl-CoA for the tricarboxylic acid (TCA) cycle to generate the adenosine triphosphate (ATP) when large amounts of energy are required. Previous research has demonstrated that acute cold exposure can affect glucose metabolism in mice's liver [14]. However, the response of glucose and lipid metabolism to chronic cold exposure in growing pigs has not yet been reported, either during cold or non-cold adaptation. Carbohydrate, an energy-supplying substance in diets, is hydrolyzed by digestive enzymes in the small intestine into monosaccharides that are more readily absorbed, like glucose. Glucose is one of the simple carbohydrates. Highly hydrophilic glucose and cannot travel freely across hydrophobic biofilms, transmembrane are required for glucose to enter cells [15]. Sodium-dependent glucose transporters (SGLTs) can actively transport glucose against a concentration gradient [16]. Moreover, sodium-independent glucose transporters (Gluts) transfer glucose along a concentration gradient [17]. Gluts are widely distributed in various animals' tissues and control the glucose transport rate in peripheral tissues and organs [18]. These glucose transporters are vital in maintaining blood glucose stability. Glucose is the energy substrate of the glycolytic system. The glucose and lipid metabolism regulation during cold exposure by dietary glucose supplementation is worth exploring.

Recent authoritative evidence suggests that gut microbiota is a vital factor that orchestrates energy homeostasis during cold exposure via regulating the gut-liver-brown fat axis and improving insulin sensitivity [19, 20]. Undoubtedly, the microbiota regulates metabolic programs during cold exposure. Based on the available research, whether the microbiome under cold exposure is represented inconsistently in different models remains unclear. The cold-adapted microbiota in healthy mice achieved higher Firmicutes versus Bacteroidetes ratios than the microbiota of mice living in warm environments, and this characteristic microbiota favors dietary energy uptake [19]. However, cold exposure decreases the ratio of Firmicutes to Bacteroidetes in obese mice caused by a high-fat diet to limit energy uptake [12]. Thus, during cold exposure, gut microbiota's host glucose and lipid metabolism regulation is complex and variable. The interaction between the gut microbiota and colonic mucosal immunity in pigs during cold and non-cold adaptation remains unknown.

Min pigs (native pig species of northern China) are renowned for their cold resistance and better gut microbiota and immunity than Yorkshire pigs [21–23]. This study aimed to investigate the regulatory effects of cold exposure on glucose and lipid metabolism, differential changes in gut microbiota and colonic mucosal immunity of Min pigs (cold-adapted) and Yorkshire pigs (non-cold-adapted).

## Materials and methods

### Cold- and non-cold-adaptive pig models establishment

**Fig. 1:**
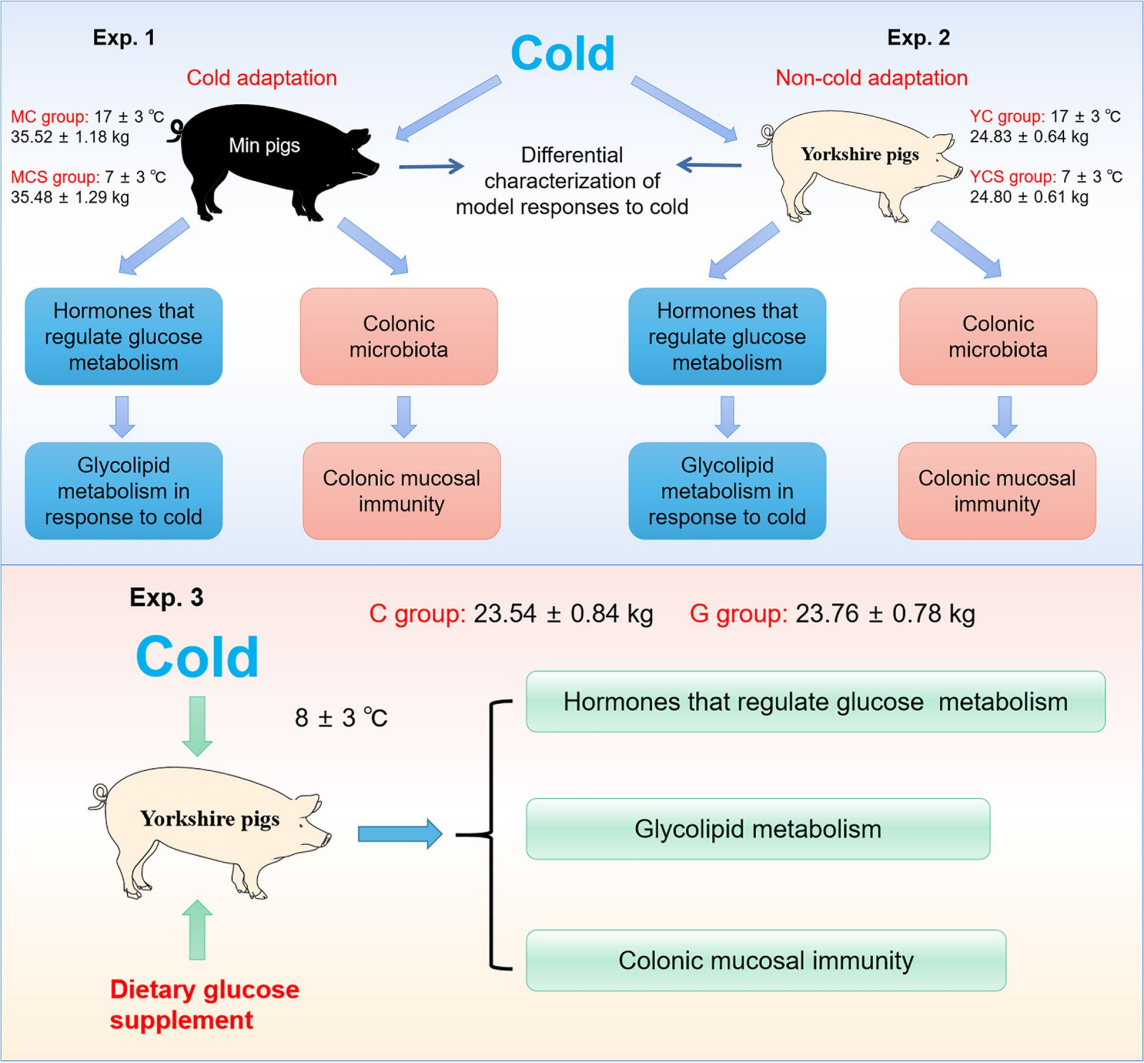
Effects of cold exposure on glucose and lipid metabolism, colonic microbiota and colonic mucosal immunity in Min pigs and Yorkshire pigs

In this study, female Min pigs (Exp. 1) were randomized into the warm control group (MC, *n* = 6) and cold exposure group (MCS, *n* = 6). Similarly, female Yorkshire pigs (Exp. 2) were also randomized into the warm control group (YC, *n* = 6) and cold exposure group (YCS, *n* = 6). These two experiments were simultaneously performed under the same experimental conditions (Fig. 1). We used electronic heaters from the Guangzhou Rongce Electronics company (GSM501, Rongce, Guangdong, China) to sustain the environment temperature of the MC and the YC group. Moreover, the environmental temperature of the MCS and YCS group was determined by the natural condition in winter (the actual detected temperature in the piggery). Pigs were individually housed in stainless steel metabolism cages (1.78 m × 0.84 m × 1.40 m), equipped with a water tank and feed trough. Water and feed were freely available throughout the rearing and experimental period. The experimental period lasted for 3 weeks. The diets (mash) in this research were performed (Table S1) with the recommendations of the National Research Council (NRC, 2012) [24]. Min pigs were purchased from the pig breeding farm in Lanxi County, Heilongjiang Province. Yorkshire pigs were purchased from the Grain Farm Group Co., Ltd.

### Treatment of dietary glucose in cold-exposed pigs

Cold-exposed female Yorkshire pig models were created in the Exp. 3 (Fig. 1). Yorkshire pigs were employed and divided into two dietary treatments: low temperature control group (C, *n* = 6) and low temperature with the glucose supplement diet group (G, *n* = 6). The diets (mash) involved in Exp. 3 were formulated (Tables S1 and S2) according to the NRC (2012) [24]. The experimental period lasted for 22 d. Pigs were individually housed in stainless steel metabolism cages (1.78 m × 0.84 m × 1.40 m), equipped with a water tank and feed trough. Water and feed were freely available throughout the rearing and experimental period. Yorkshire pigs were purchased from the Grain Farm Group Co., Ltd.

The Animal Care and Use Committee at Northeast Agricultural University in Heilongjiang Province, China (NEAU—[2011]—9) has examined and approved all experimental animal protocols covering the care and management of animals. The animal care and treatment methods complied with Heilongjiang Province's "Laboratory Animal Management Regulations" (updated 2016).

### Sample collection

All pigs were sacrificed by electric shock after fasting for 12 h. Venous blood was obtained, and stored in a heparin sodium tube. Then, the plasma was assembled by centrifugation and stored at −40 °C. About 4 g of livers (median lobe), longissimus dorsi muscles (middle part), and leg muscles (middle part) were quickly collected in the cryo-storage tube. The middle colonic segment's contents were collected into sterile cryo-storage tubes and immediately placed in liquid nitrogen to identify colonic microbiota. The glass slide collected mucosal scrapings of the jejunal mucosa (front section) and colon (middle section). These tissue samples mentioned above were transferred to the ultra-low temperature refrigerator for storage until use.

About 1 cm^3^ of the colon was preserved in a 10% formaldehyde solution. Colon sections were stained with hematoxylin and eosin (H&E) for histological structural studies and immunohistochemistry. Then, another 1 mm^2^ of colon tissue was obtained in an electron microscopic solution at 4 °C. The ultrastructure of colon tissue was analyzed by using an electron microscope.

### Plasma glucose and hormones

The plasma glucose was assessed with an automated biochemical analyzer (Synchron CX® 4 Pro Beckman Coulter, Brea, CA, USA) by commercial diagnostics kits (Biosino Bio-Technology and Science Inc., Beijing, China). After determining the total protein concentration in plasma by the BCA Protein Assay kit (Shanghai Beyotime Biotechnology, China), insulin, cortisol, T3 and T4 in plasma were quantified by radioimmunoassay (Beijing Huaying Institute of Biotechnology, Beijing, China). An ELISA kit from Enzyme Biosystems (Shanghai, China) was used to detect plasma glucagon levels. This procedure was conducted strictly according to the instructions through the enzyme label analyzer (Labsystems Multiskan MS, Amersham Bioscience Ltd., Little Chalfont, UK).

### Enzymes associated with glucose metabolism

About 1 g liver sample was taken from −80 ºC and mixed with 9 mL of physiological saline. After 5 min of homogenization (5,000 × *g*), and 10% tissue homogenate was prepared and then centrifuged at 3,500 × *g* for 10 min. The BCA Protein Assay kit from Beyotime Biotechnology (Shanghai, China) was employed to evaluate the total protein concentration. Then, the ELISA kit from Enzyme Biosystems (Shanghai, China) was employed to evaluate the concentration of hexokinase (HK), pyruvate kinase (PK), pyruvate carboxylase (PC), phosphoenolpyruvate kinase (PEPCK) and glucose-6-phosphate kinase (G6PC) in the liver.

### Key enzymes activity in glycogen metabolism

Glycogen phosphorylase (PYGL) and glycogen synthase (GS) activity in the liver was detected using the kit from Enzyme Biosystems (Shanghai, China) according to the manufacturer's instructions.

### Histopathology and ultrastructure analysis

Colon samples were fixed with 10% paraformaldehyde and then embedded in paraffin. They were sectioned into thin slices and stained with the H&E. Next, these sections were examined through the Nikon Eclipse Ci-L microscope (Nikon, Tokyo, Japan) at 200 × magnification. All images in this study were captured by the Nikon DS-F12 digital camera (Nikon, Tokyo, Japan).

After being treated in 2.5% glutaraldehyde, colon samples were rinsed and fixed with osmium tetroxide. Then they were dehydrated with ethanol and acetone. Next, they were embedded. The ultramicrotome was then used to cut ultrathin sections stained with uranyl acetate and lead citrate. The sample ultrastructure was examined using Transmission Electron Microscope (TEM) (Hitachi H-7650, Tokyo, Japan).

### Quantitative RT-PCR analysis

TRIzol Reagent manufactured by the Takara Biomedical Technology company (Dalian, Liaoning, China) was used to extract total RNA from samples. Next, the total RNA was reverse transcribed into cDNA using PrimeScript™ RT reagent kit with gDNA Eraser kit (Takara Biomedical Technology Co., Ltd., Dalian, Liaoning, China). RT-qPCR was conducted by the SYBR Green mix (Takara Biomedical Technology Co., Ltd., Dalian, Liaoning, China) to assess the mRNA expression. The relative mRNA expression of the target gene was normalized to the β-actin and defined [22] using the 2^–ΔΔCt^. Information on all the primers is summarized in Table S3.

### Western blot analysis

The total protein from samples was extracted with the RIPA buffer (including 1% PMSF). The protein concentration was assessed by a BCA Protein Assay kit from Beyotime Biotechnology. Total protein was transferred to the polyvinylidene fluoride (PVDF) membrane after sodium dodecyl sulfate–polyacrylamide gel electrophoresis (SDS-PAGE). Next, membranes were immunoblotted with the indicated primary antibodies for 14 h at 4 ºC after blocking with 5% nonfat milk for 2 h. They were then incubated with the HRP-conjugated secondary antibody for 2 h at 25 ºC. Finally, the signal detection of membranes was performed using ECL Substrate (Beyotime, Shanghai, China). ImageJ software was utilized to represent band intensity. The relative intensity of target proteins was normalized by the band values of β-actin. Table S4 displayed the information about antibodies involved in this study.

### Metabolomic profiling

Briefly, 100 µL plasma was combined with 300 µL methanol/water mixed solvent (0.1% formic acid, v/v). Next, the mixture was homogenized. After 15 min of ultrasonic in 20 ºC water baths, vortex for 2 min, and centrifugation at 12,000 × *g* for 20 min at 4 ºC, the supernatant was taken. Moreover, the machine (Quadrupole time-of-flight liquid-mass system, Agilent Technology Co., Ltd., Beijing, China) was used for detection. Agilent Profinder software was used to conduct retention time correction, peak recognition, peak extraction, peak integration, peak alignment and other work on the original MS data, and then CEF files were generated. Then the Agilent Massive Parallel Processor software was used for statistical processing, and the associated Metlin database was used for substance identification (Bioacme Coa, Wuhan, Hubei, China). Heatmaps of differential metabolites were drawn in R with heatmap package version 1.0.12. The values of variable-importance projection > 1 and *P* < 0.05 were considered significantly different.

### 16S rDNA gene sequencing analysis

QIAamp DNA Stool Mini kit (Qiagen, Hilden, Germany) was used to extract the total bacterial DNA from colonic contents (*n* = 6). The V4 hypervariable region of the 16S rDNA gene was PCR-amplified via the primers 515F and 806R. Paired-end sequencing was carried out on an Illumina HiSeq 2500 platform (Bioacme Coa, Wuhan, China). Raw reads in this study were filtered and merged as raw tags using the FASTP. After that, raw tags involved in this study were further filtered to produce clean tags. Then, clean tags were used for clustering to get operational taxonomic units (OTUs) after quality filter. Then, the abundance of OTU was conducted. The raw data involved in this research has been shared in public databases (https://www.ncbi.nlm.nih.gov/sra/PRJNA886839).

### Short chain fatty acids

We mixed 1 g of the colonic digesta with 1 mL of ultrapure water. Then, the well-mixed samples were centrifuged at 10,000 × *g* for 10 min at 4 °C. Next, the supernatant was filter-sterilized with a 0.22-μm filter (this step was repeated three times), and absorbed into the sampling bottle for GC–MS/MS analysis, as described in the previous study [25].

### Statistical analysis

Firstly, the data in this study were integrated and calculated using Microsoft Excel 2019. The individual pig was considered as the experimental unit. Then, we evaluated the normality and homogeneity of variances of the data in this research. Next, the significance of differences was analyzed by “*t*-test” in SPSS 25.0 (IBM-SPSS Inc, Chicago, IL, USA). The data were visualized by the GraphPad Prism (version 8.0.2, Graph Pad Software Inc., San Diego, CA, USA). Data in this research are expressed as mean ± SEM. Differences were considered significant when *P* < 0.05. “*” means *P* < 0.05, “**” means *P* < 0.01. The programming language R (version 4.2.1, The Comprehensive R Archive Network, USA) was used for correlation analysis involved in this research.

## Results

### Cold exposure is associated with hormones that regulate glucose homeostasis and glucose metabolism

**Fig. 2:**
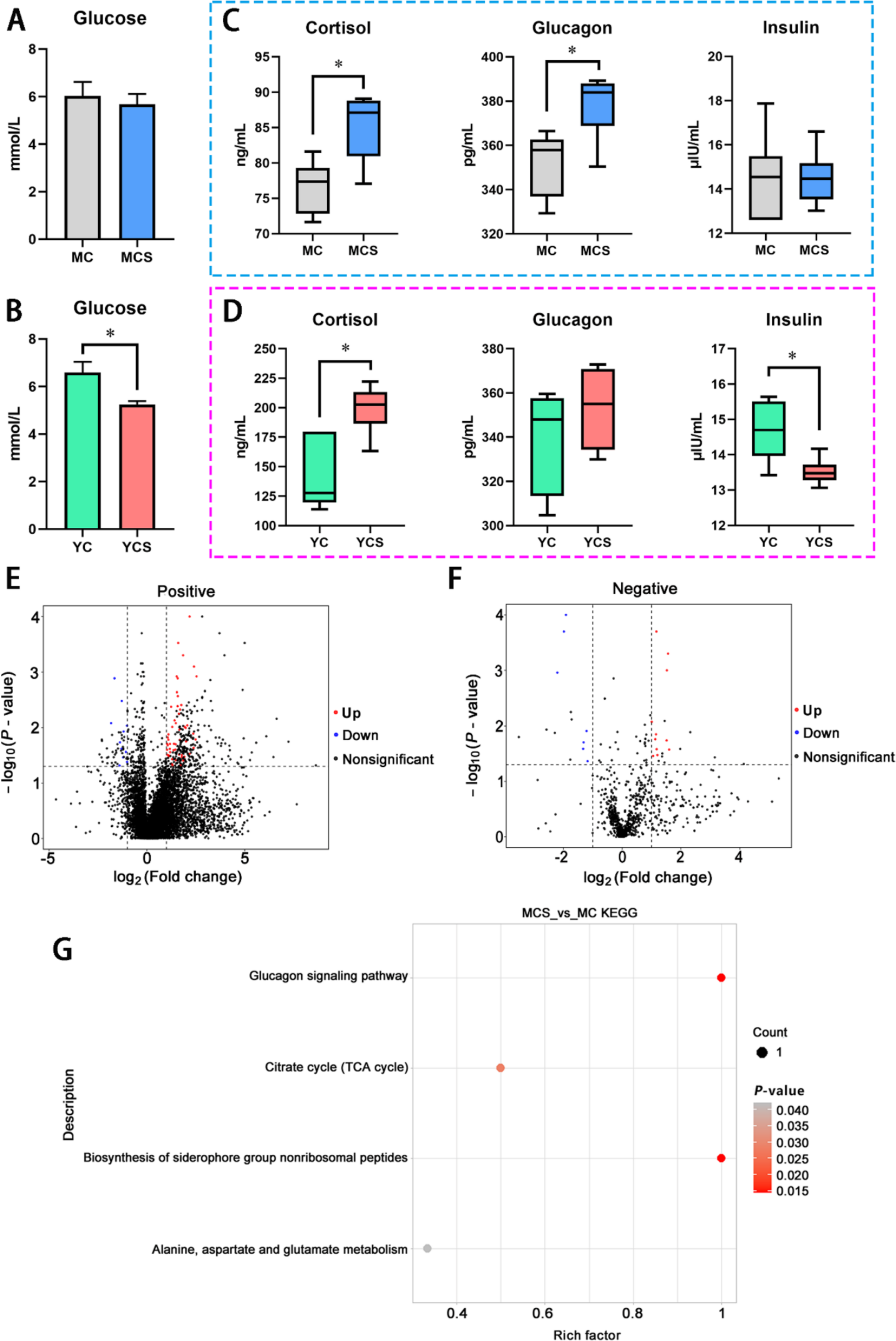
Development of cold-adaptive and non-cold-adaptive models, and plasma metabolic parameters during cold exposure. **A** Plasma glucose of Min pig models. *n* = 6. **B** Plasma glucose of Yorkshire pig models. *n* = 6. **C** Plasma hormones in Min pig models. *n* = 6. **D** Plasma hormones in Yorkshire pig models. *n* = 6. **E** Volcanic map of metabolic differences in positive ion mode of Min pigs. *n* = 5. **F** Volcanic map of metabolic differences in negative ion mode of Min pigs. *n* = 5. **G** KEGG analysis of plasma metabolites of Min pigs. ^*^P < 0.05. Other factors without significant changes are shown in Fig. S1

We first evaluated the establishment of cold- and non-cold-adaptive models. Intriguingly, adaptive phenotypes were identified in Min pig models, which had thicker fur to better adapt to the cold (Fig. S1A). This phenomenon was not observed in the Yorkshire pig model (Fig. S1B). Moreover, plasma glucose remained stable during cold exposure in Min pig models, but decreased in Yorkshire pigs (Fig. 2A and B). Then, we characterized the glucose-metabolism-related hormone concentrations of both models during the cold. In Min pig models, insulin, T3 and T4 levels were unchanged, whereas the concentrations of plasma cortisol and glucagon were increased by cold exposure (Fig. 2C, Fig. S1C and D). In Yorkshire pig models, cold exposure inhibited the insulin concentrations in Yorkshire pigs, accompanied by an increase in cortisol concentrations (Fig. 2D). These data suggested that cold exposure promoted the release of stress-related hormones. Notably, although the drop in plasma insulin levels in the YCS group favored the glucose generation route, low plasma glucose levels were continuously observed. However, stable plasma glucose levels were detected in the MCS group, accompanied by increased in glucagon concentration.

Next, the plasma metabolic profiles of the two pig models were characterized. For the positive-ion mode, 55 metabolites were significantly up-regulated and 11 were down-regulated in the MCS group. For the negative-ion mode, 12 were up-regulated and 7 were down-regulated (Fig. 2E and F, Table S5). KEGG revealed that the MCS group was abundant in the glucagon signaling pathway, citrate cycle (TCA cycle), biosynthesis of siderophore group nonribosomal peptides, and amino acid metabolism pathway (Fig. 2G). Notably, glucagon signaling pathway activation suggested that the glucagon signaling pathway might be vital in maintaining glycemic homeostasis during cold adaptation. In Yorkshire pig models, 66 were significantly up-regulated for positive-ion mode and 64 down-regulated in the YCS group. For the negative-ion mode, 15 were up-regulated and 1 was down-regulated (Fig. S1G and H, Table S6). KEGG revealed that the YCS group was abundant in the ketone body synthesis and degradation pathway (Fig. S1I).

### Cold exposure causes excessive glucose consumption

The glucose in diets is mainly absorbed in the small intestine. We found that the mRNA expression of sodium glucose co-transporter (*SGLT1)* in jejunal mucosa was increased in the MCS group (Fig. S2A). Yorkshire pigs' jejunal mucosa contained no glucose transporter alterations (Fig. S2B). Thus, under the same cold exposure conditions, the small intestine of Min pigs might transport more glucose in the intestinal lumen.

**Fig. 3:**
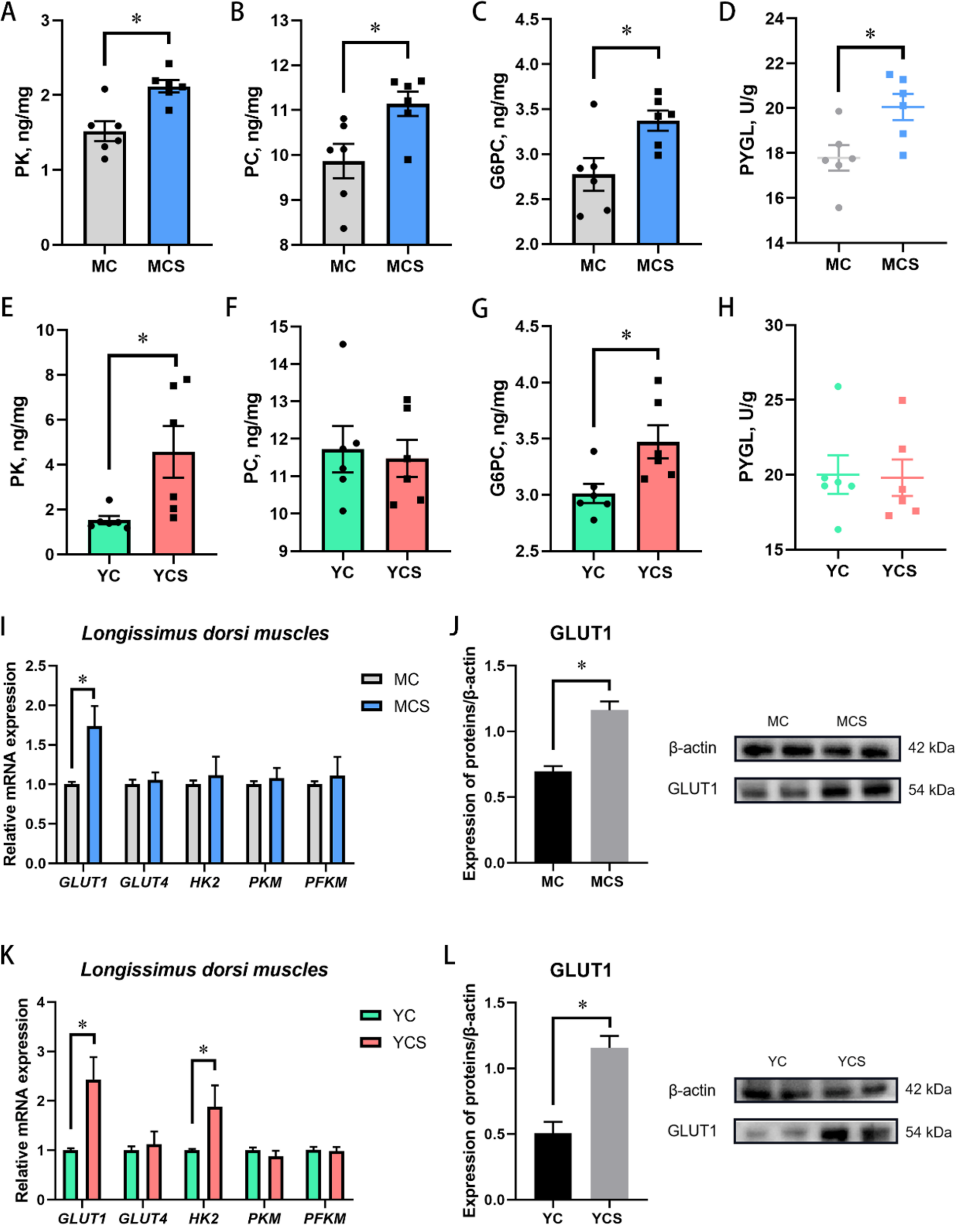
Large amounts of glucose are consumed during cold exposure. **A**–**C** Pyruvate kinase (PK), pyruvate carboxylase (PC) and glucose-6-phosphate kinase (G6PC) concentration in the liver of Min pigs. *n* = 6. **D** Glycogen phosphorylase (PYGL) activity in the liver of Min pigs. *n* = 6. **E**–**G** PK, PC and G6PC concentration in the liver of Yorkshire pigs. *n* = 6. **H** PYGL activity in the liver of Yorkshire pigs. *n* = 6. **I** Glucose transport and glycolysis in the longissimus dorsi muscle of Min pigs. *n* = 6. **J** GLUT1 protein expression in the longissimus dorsi muscle of Min pigs. *n* = 4. **K** Glucose transport and glycolysis in the longissimus dorsi muscle of Yorkshire pigs. *n* = 6. **L** GLUT1 protein expression in the longissimus dorsi muscle of Yorkshire pigs. *n* = 4. ^*^P < 0.05. Other factors without significant changes are shown in Fig. S2

Livers are the central nexus of energy metabolism in mammals. The dynamic equilibrium of glycogen metabolism, glycolysis, and gluconeogenesis ensures the constant replenishment of ATP and glucose homeostasis. We explored differences in glucose metabolism in the liver of cold-adapted and non-cold-adapted models driven by cold exposure. In Exp. 1, higher concentrations of PK, PC and G6PC were detected in the liver of the MCS group than in the MC group (Fig. 3A–C). Cold exposure did not alter GS activity, HK and PEPCK concentrations in the liver of the MCS group (Fig. S3A–C). Both glycolysis and gluconeogenesis were stimulated by cold exposure in Min pig models. Surprisingly, the PYGL activity was elevated in the MCS group (Fig. 3D). The hepatic glycogen breakdown fueled the ongoing glucose consumption under cold exposure.

In Yorkshire pig models, PK and G6PC concentrations also increased in the YCS group (Fig. 3E and G). Other enzymes associated with glucose metabolism were not altered (Fig. 3F and Fig. S3D–F). Notably, cold exposure promoted glycolysis and gluconeogenesis pathways in the liver of both pig models. According to these findings, low temperatures expedited the glucose breakdown to provide a constant supply of ATP. Gluconeogenesis promotion is an active adaptation to maintain glucose homeostasis during cold exposure.

Peripheral muscle and adipose tissue protect the body during cold exposure. We characterized the glucose transport and utilization in the blood by the muscle and adipose tissue. The results showed that cold exposure did not affect glucose transporter 4 (*GLUT4)* expression of the MCS and YCS group in the dorsal fat (Fig. S3G and I). Glucose utilization and turnover of the leg muscle were also not affected in the MCS and YCS group (Fig. S3H and J). However, we found GLUT1 mRNA and protein expression were increased in both MCS and YCS groups (Fig. 3I–L). Hexokinase type 2 (*HK2)* mRNA expression was enhanced in the YCS group (Fig. 3K). The glycolysis pathway of longissimus dorsi muscles was more active in the YCS group, indicating that the muscle tissue of Yorkshire pigs needed more glucose during cold exposure.

### Cold exposure interferes with the gut microbiota during cold adaptation and non-cold adaptation

**Fig. 4:**
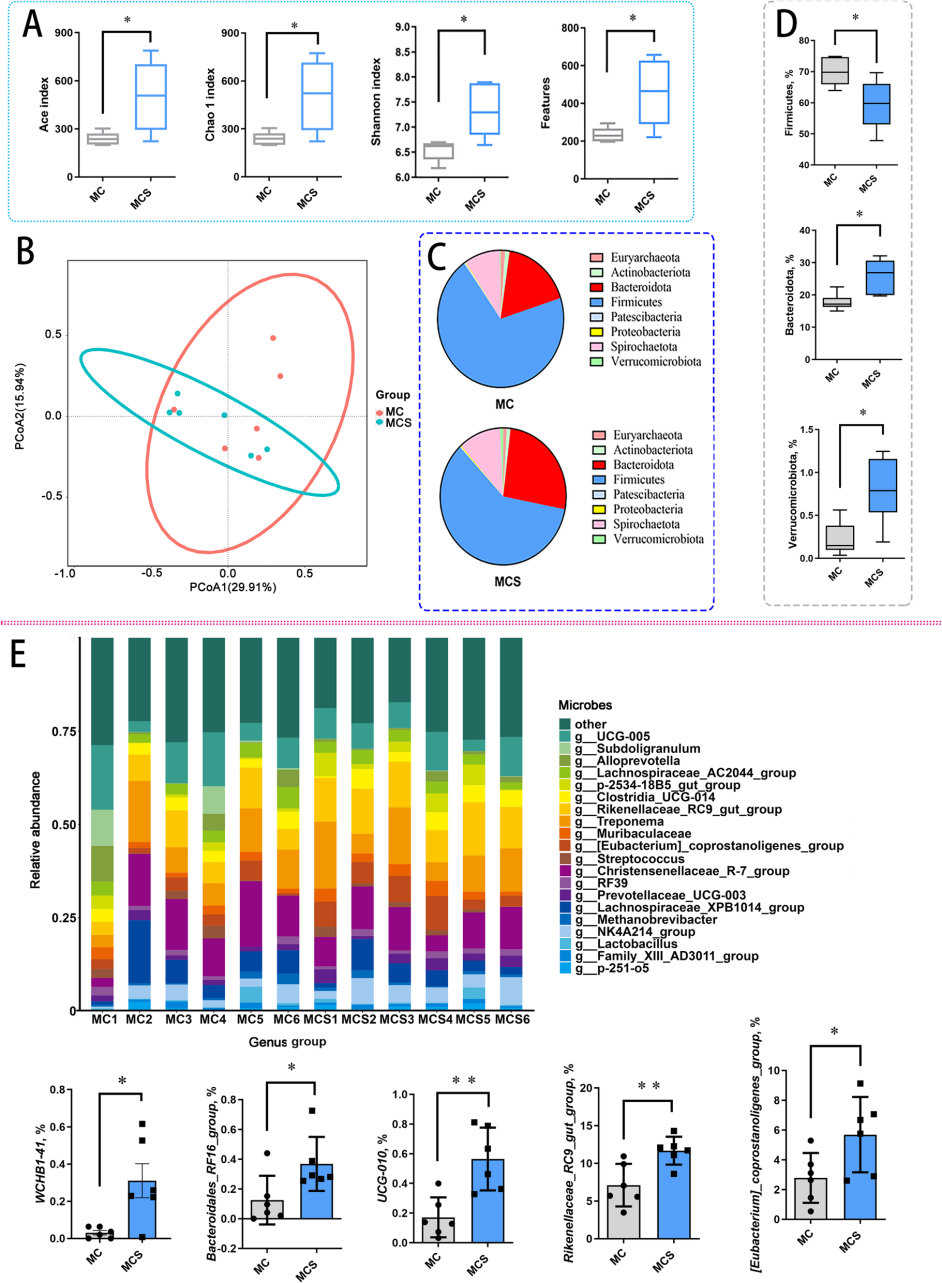
Effects of chronic cold exposure on colonic microbiota of Min pigs. **A** Colonic microbiota diversity index of Min pigs. **B** PCoA analysis of colonic microbiota in Min pigs. **C** Phylum level of colonic microbiota in Min pigs. **D** Firmicutes, Bacteroidetes and Verrucomicrobiota abundance in Min pigs. **E** Genus level of colonic microbiota in Min pigs. *n* = 6. ^*^P < 0.05. ^**^P < 0.05

The gut microbiota modulates immunological homeostasis and metabolic regulation in mammals. In two models, we focused on changes in the colonic microbiota during cold exposure. Cold alters colonic microbiota with increased microbial diversity and richer features (Fig. 4A). Gut microbiota in the MCS group was characterized by a relatively higher abundance of Bacteroidetes and Verrucomicrobiota, while a lower abundance of Firmicutes (Fig. 4C and D). Then, we sought to identify changes at the genus level. *Rikenellaceae_RC9_gut_group*, *[Eubacterium]_coprostanoligenes_group*, *UCG-010*, *Bacteroidales_RF16_group* and *WCHB1-41* abundance were increased in the MCS group (Fig. 4E). Notably, cold exposure improved valeric acid and isovaleric acid concentrations in colonic contents of the MCS group, providing energy substrates for colonic epithelial cells (Fig. S4A and B).

**Fig. 5:**
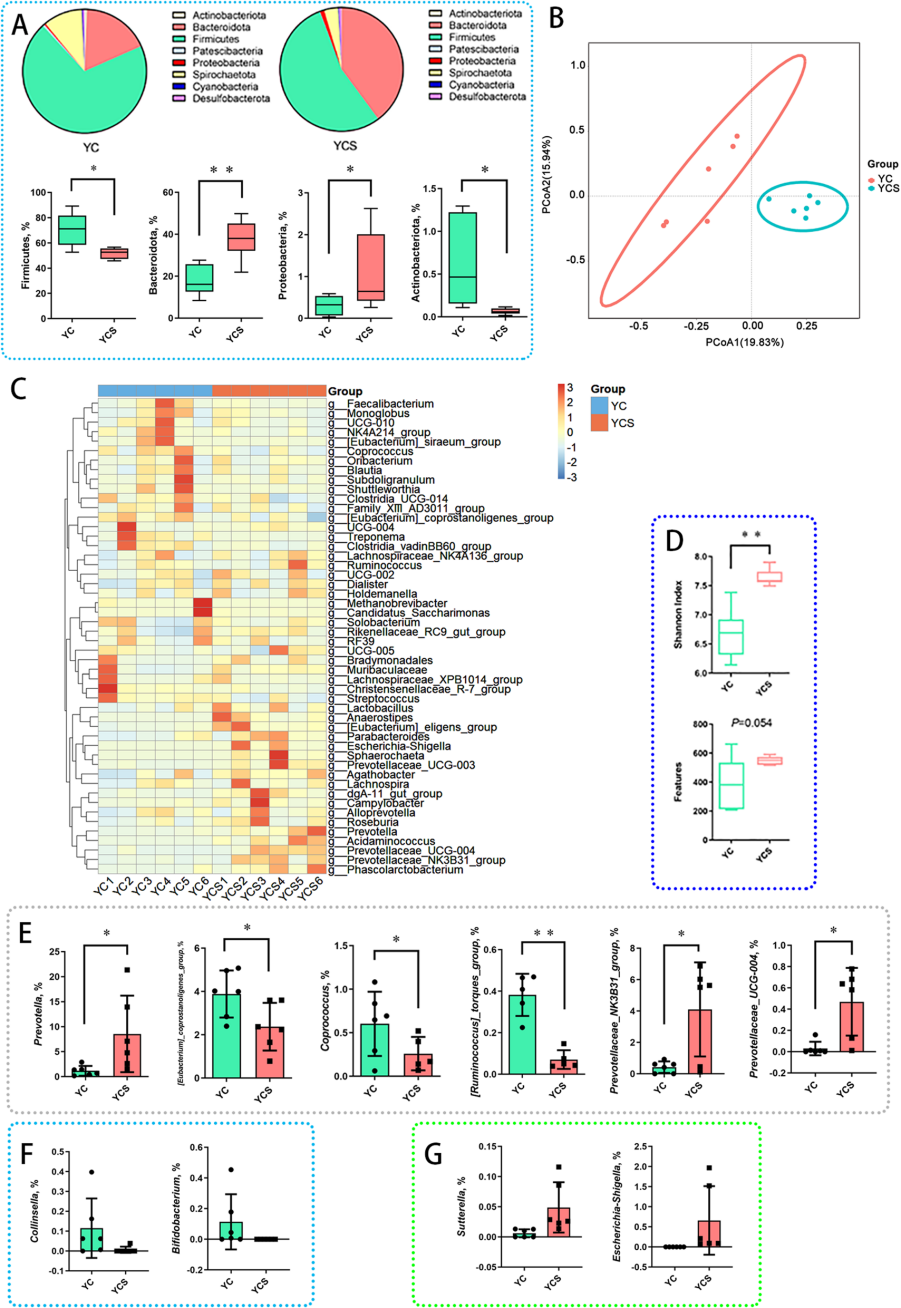
Effects of chronic cold exposure on colonic microbiota of Yorkshire pigs. **A** Phylum level of colonic microbiota in Yorkshire pigs. **B** PCoA analysis of colonic microbiota in Yorkshire pigs. **C** Heat map of genus level abundance. **D** Colonic microbiota diversity index of Yorkshire pigs. **E** Genus level of colonic microbiota in Yorkshire pigs. **F** Two genera depletion in Actinobacteria of Yorkshire pigs. **G** Two genera of Proteobacteria are enriched. *n* = 6. ^*^P < 0.05, ^**^P < 0.01

For the Yorkshire pig model, PCoA analysis showed that the Yorkshire pig microbiota was regulated by cold exposure (Fig. 5B). An increase in the Shannon index was detected in the YCS group (Fig. 5D). Similarly, we characterized the dominant phyla of Yorkshire pigs. The changes in Firmicutes and Bacteroidetes abundance in the Yorkshire pig model were similar to the Min pig model. However, notably, a higher abundance of Proteobacteria and a lower abundance of Actinobacteriota was characterized in the YCS group (Fig. 5A). We further analyzed the top 50 genus levels. *Prevotella*, *Prevotellaceae_NK3B31_group* and *Prevotellaceae_UCG_004* abundance was increased, while *Coprococcus* was decreased in the YCS group. Intriguingly, *[Ruminococcus]_torques_group* and *[Eubacterium]_coprostanoligenes_group* abundance was reduced in the YCS group (Fig. 5E). We also focus on several representative genera of Actinobacteriota. *Collinsella* and *Bifidobacterium* abundance was depleted (Fig. 5F). Moreover, in Proteobacteria, *Sutterella* and *Escherichia-Shigella* abundance was enriched in the YCS group (Fig. 5G). We could not detect changes in the concentration of SCFAs in colon contents of Yorkshire pig models during cold exposure (Fig. S4C and D). Particularly, the key members (*Collinsella* and *Bifidobacterium*) of Actinobacteriota were depleted during the cold, which was widely regarded as probiotics. Compared to the YC group, the YCS group had more *Sutterella* and *Escherichia-Shigella* in the colonic contents.

### Cold exposure induced colonic mucosal barrier injury and promoted mitophagy

Gut microbes regulate host immunity and metabolism. Based on cold-induced differences in the microbiota of the two pig models, we further analyzed colonic mucosal function and immunity during cold exposure. The H&E section and ultrastructural analysis reveal no pathological changes in Min pig models (Fig. S5A). Intriguingly, the expression of porcine β-defensin 2 (*PBD-2*) and *PR-39* was enhanced in the MCS group's colonic mucosa than in the MC group (Fig. S5B). The colonic mucosal barrier and mitochondrial function of the Min pig models were almost not altered by cold (Fig. S5C and S5D).

**Fig. 6:**
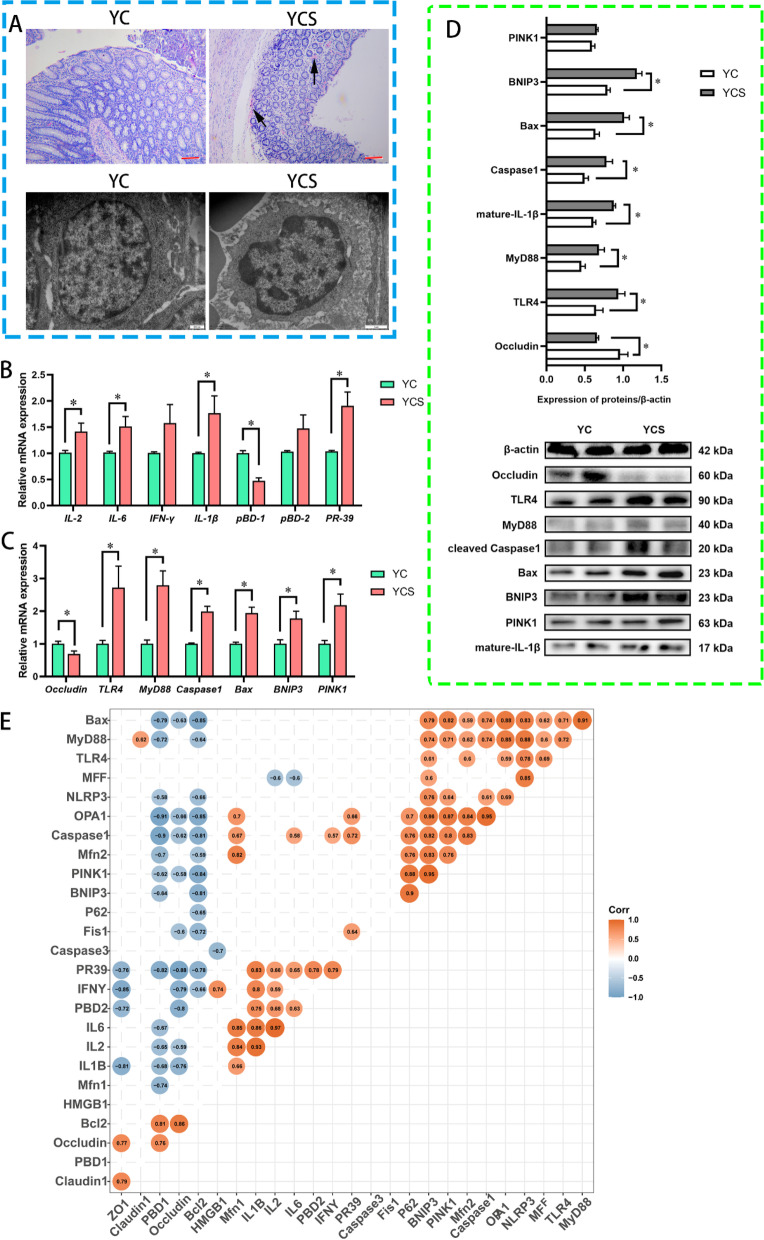
Chronic cold exposure induces colonic barrier injury and mitochondrial dysfunction in Yorkshire pigs. **A** Pathological sections and ultrastructure of the colon of Yorkshire pigs. The arrows show the infiltration of inflammatory cells. **B** The mRNA expression of inflammatory cytokines and antimicrobial peptides in colon mucosa of Yorkshire pigs. *n* = 6. **C** The mRNA expression of tight junction proteins, inflammatory pathways, apoptosis and mitophagy related genes in colon mucosa of Yorkshire pigs. *n* = 6. **D** Protein expression in the colon mucosa of Yorkshire pigs. *n* = 4. **E** Correlation coefficients between mucus layer, inflammatory response, and epithelial barrier. Correlation coefficients > 0.5 or ≤ 0.5. Orange and blue colors denote positive and negative correlations, respectively. Color intensity is proportional to Spearman’s rank correlation values. *n* = 6. ^*^P < 0.05. Other factors without significant changes are shown in Fig. S4

However, a small quantity of inflammatory cell infiltration was detected in the colon of Yorkshire pigs, and the nucleus structure was disrupted (Fig. 6A). Examination of the physical barrier and immune factors of the colonic mucosa revealed that the Occludin and porcine β-defensin 1 (*pBD-1)* expression was suppressed in the Yorkshire pig models with higher mRNA expression of *IL-1β*, *IL-2*, and *IL-6*, although the *PR-39* was enhanced by cold (Fig. 6B). Obviously, cold exposure increased the risk of colon inflammation, which was most likely due to non-cold-adapted microbiota, as the physical barrier and inflammatory factors of the colon in Min pigs were not regulated by the cold. We further analyzed the classical inflammatory pathways and apoptosis in the colonic mucosa. The expression of TLR4, MyD88, Caspase1 and bcl-2- associated X-protein (Bax) was all promoted by cold exposure in the YCS group (Fig. 6C and D). These results indicated that TLR4/MyD88 pathway and apoptosis were enhanced in the Yorkshire pig models. Mitochondria generate ATP through oxidative phosphorylation. Cold-induced energy expenditure motivated us to focus on the mitochondrial function in the colonic mucosa. We observed that the expression of bcl-2/adenovirus E1B 19-kDa interacting protein (*BNIP3)*, PTEN induced kinase 1 *(PINK1)*, mitofusin 1 (*Mfn1*), mitofusin 2 (*Mfn2*), and optic Atrophy 1 (*OAP1*) in the colonic mucosa of the YCS group was increased by cold exposure (Fig. S5E–G). These data suggested that cold exposure enhanced mitophagy and mitochondrial fusion in non-cold-adapted models' colonic mucosa. A correlation test by taking the cytokines, tight junction protein, apoptosis, mitophagy and mitochondrial kinetic equilibrium utilization was employed to investigate the correlation between the mucosal immunity and mitochondrial function in Yorkshire pigs during the cold (Fig. 6E). We found that the expression of tight junction proteins was negatively correlated with the apoptosis, mitophagy, mitochondrial fusion, and proinflammatory cytokines. Moreover, the proinflammatory pathway was significantly correlated with the apoptosis and mitophagy. We discovered that cold exposure did not affect colonic mucosal immunity and mitochondrial function in Min pig models. In contrast, Yorkshire pig models exhibited an increase in the inflammatory pathway, apoptosis, mitophagy, and mitochondrial fusion.

### Cold-induced glucose overconsumption promotes lipolysis

**Fig. 7:**
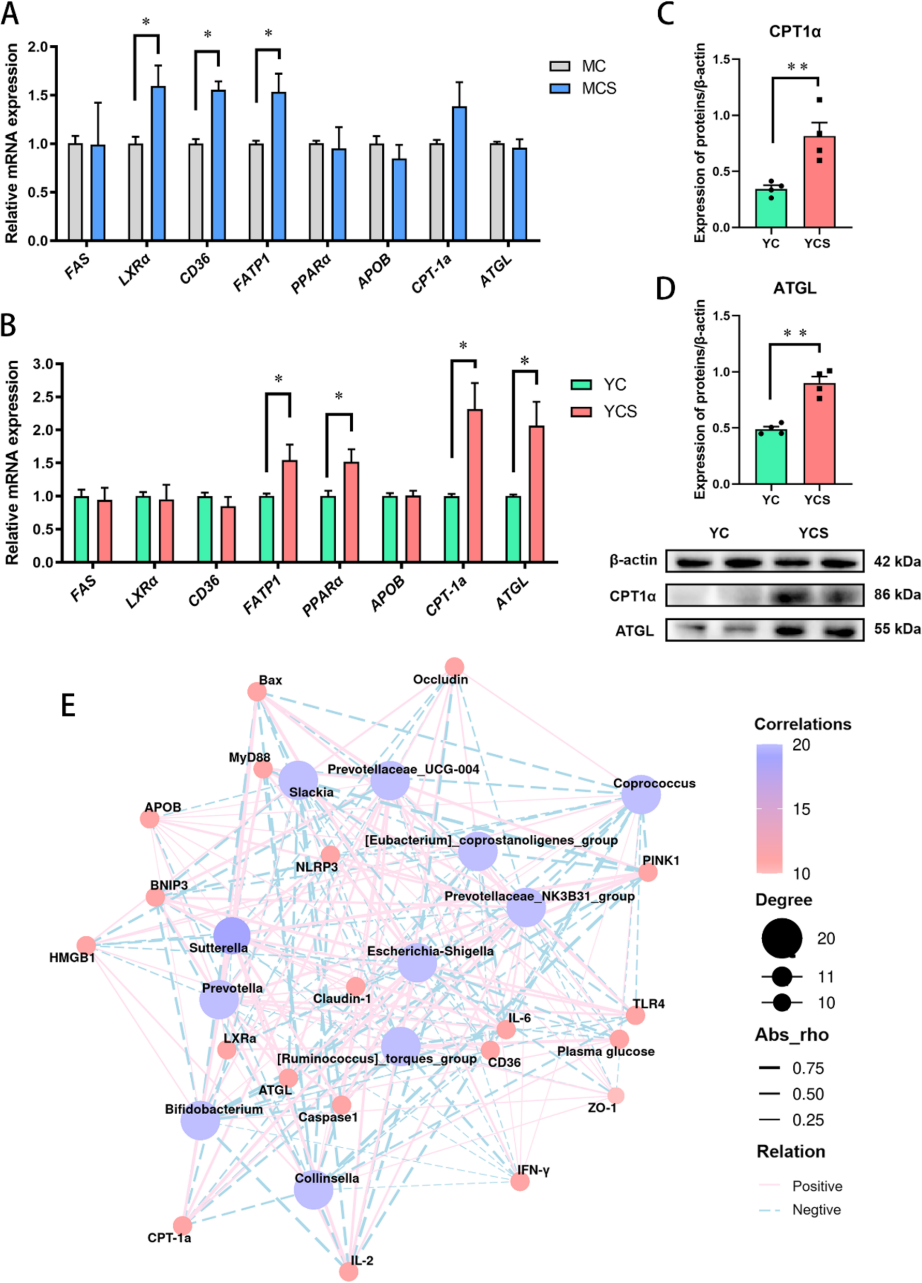
Excessive glucose consumption induced by cold exposure promotes lipolysis and fatty acid oxidation. **A** The mRNA expression of fat metabolism related genes in the liver of Min pigs. **B** The mRNA expression of fat metabolism related genes in the liver of Yorkshire pigs. **C** and **D** CPT-1α and ATGL protein expression in the liver of Yorkshire pigs. **E** Correlation coefficients between plasma glucose, mucus layer, inflammatory response, epithelial barrier and microbiota. Correlation coefficients > 0.5 or ≤ 0.5. Pink and blue colors denote positive and negative correlations, respectively. Color intensity is proportional to Spearman’s rank correlation values. *n* = 6. ^*^P < 0.05. ^**^P < 0.01

KEGG analysis of plasma metabolites from Yorkshire pig models during cold exposure revealed an enrichment of ketone body-related pathways. Consequently, we must rapidly examine the reaction of fatty acid oxidation pathways to cold exposure. The outcomes indicated that the nuclear receptor liver X receptor α (*LXRα*), fatty acid transport protein cluster of differentiation 36 (*CD36*), and fatty acid transporter 1 (*FATP1*) mRNA expression in the liver of the MCS group was enhanced than the MC group (Fig. 7A). Differently, we found that the expression of peroxisome-proliferator-activated receptor alpha (*PPARα*), carnitine palmitoyl transferase 1α (CPT-1α) and adipose TG lipase (ATGL) was increased in the liver of the YCS group (Fig. 7B–D). We also examined the gene expression levels of genes related to lipid metabolism in the dorsi fat and longissimus dorsi muscle. Compared to the MC group, the mRNA expression of *CPT-1a* in the dorsal fat of the MCS group was enhanced by chronic cold exposure, but the mRNA expression of *PPARα* and transcription factor CCAAT enhancer binding protein α (*C/EBPα*) was not changed (Fig. S6A). The expressions of *CD36*, acetyl CoA carboxylase (*ACC*) and fatty acid synthase (*FAS*) mRNA in the longissimus dorsi muscle of Min pigs were also not affected (Fig. S6B). Compared with the YC group, chronic cold exposure promoted the *CPT-1a* expression in the YCS group's dorsal fat (Fig. S6C). Chronic cold exposure promoted the *FAS* expression in the longissimus dorsi muscle of the YCS group, but inhibited the *CD36* and *ACC* expression (Fig. S6D).

### Lipolysis was inhibited during cold exposure when dietary glucose was adequate

**Fig. 8:**
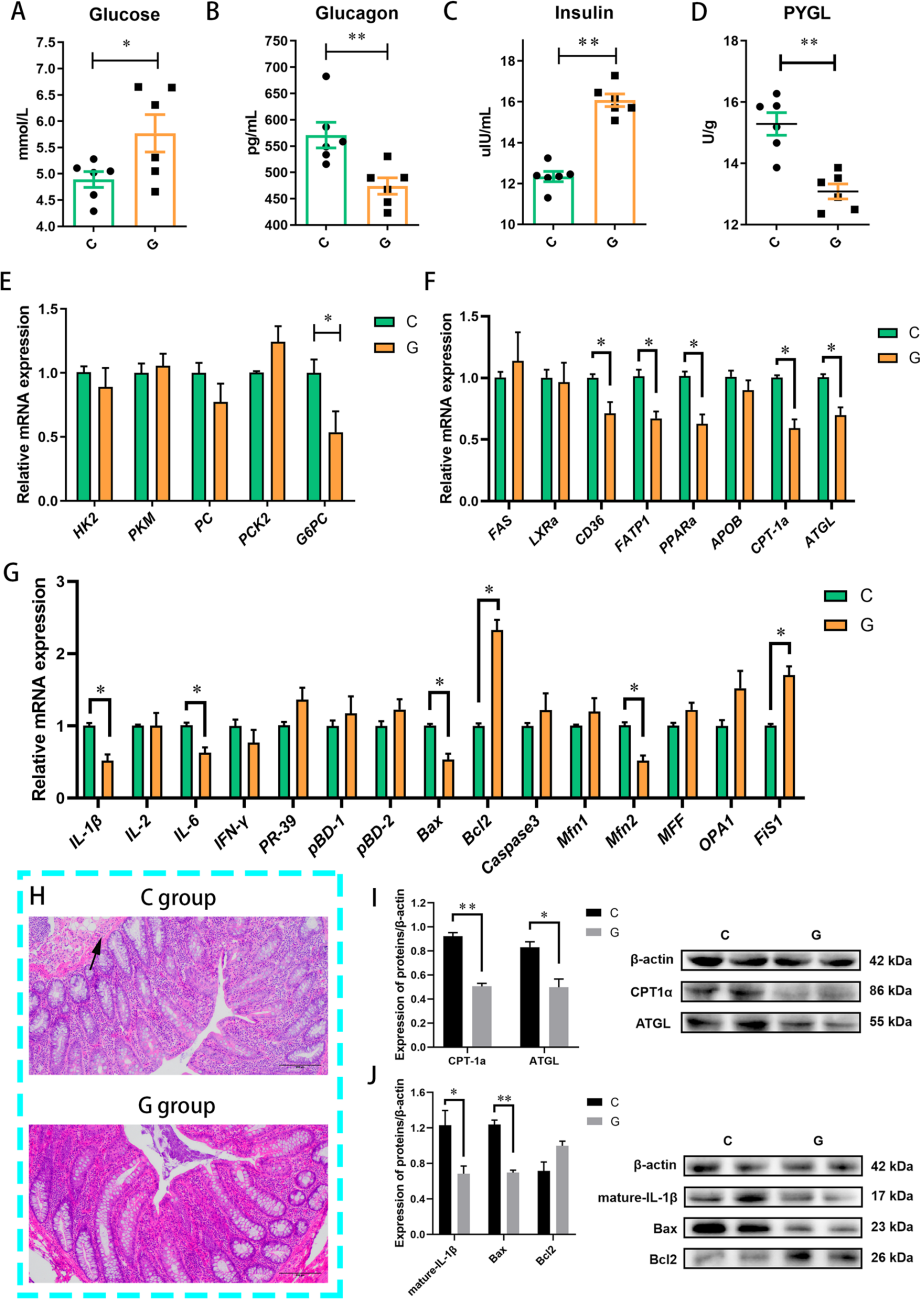
Provide adequate glucose during cold exposure regulated glucose metabolism, lipid metabolism and colonic mucosal immunity in Yorkshire pigs. **A**–**C** Plasma hormone levels, *n* = 6. **D** Glycogen phosphorylase (PYGL) activity in the liver, *n* = 6. **E** Glycolysis and gluconeogenesis in the liver, *n* = 6. **F** Fat metabolism in the liver, *n* = 6. **G** Colonic mucosal immunity and mitochondrial function, *n* = 6. **H** Pathological sections of the colonic mucosa of a Yorkshire pig (*n* = 3). **I** ATGL and CPT-1α protein expression in the liver, *n* = 4. **J** Mature-IL-1β, Bax and Bcl2 protein expression in the colonic mucosa, *n* = 4. ^*^P < 0.05, ^**^P < 0.01

Correlation analysis showed that the decreased plasma glucose in Yorkshire pig models during cold exposure had a strong correlation with factors related to intestinal mucosal injury, lipolysis and microbiota (Fig. 7E). Yorkshire pigs were fed glucose to demonstrate that high glucose consumption during cold exposure promotes lipolysis and interferes with the microbiota. As dietary glucose levels rose, plasma glucose levels were increased in the G group (Fig. 8A). To stabilize glucose homeostasis, glucagon concentration was inhibited and insulin concentration was promoted (Fig. 8B and C). As for glucose transport in the jejunal mucosa, we found that the *SGLT1* mRNA expression in jejunal mucosa of the G group was significantly enhanced compared to the C group (Fig. S7). Moreover, the PYGL activity and *G6PC* mRNA expression in the liver were inhibited (Fig. 8D and E). These results followed that the glycogenolysis and gluconeogenic pathways were restricted during cold exposure when glucose was provided in sufficient quantities. Furthermore, compared to the C group, the expression of *CD36*, *FATP1*, *PPARα*, *CPT-1α* and ATGL in the liver of the G group was down-regulated during cold exposure (Fig. 8F and I). Lipolysis was inhibited during cold exposure when dietary glucose was adequate.

### Colonic mucosal immunity was improved during cold exposure when dietary glucose was adequate

Carbohydrates are an essential energy source. As one of the typical carbohydrates, glucose is the primary energy source for cells. We found that glucose supplementation during cold exposure decreased the IL-1β and IL-6 expression in the colonic mucosa of the G group (Fig. 8G and J). Moreover, energy supplementation inhibited apoptosis, accompanied by the restraint of Bax and augmented levels of Bcl2 (Fig. 8G and J). Intriguingly, *Mfn2* mRNA expression in the colonic mucosa was inhibited while *Fis1* mRNA expression was promoted in the G group, which biased mitochondrial dynamics toward fission (Fig. 8G). According to H&E Staining Sections, inflammatory cell infiltration in the colon of the G group was not evident (Fig. 8H). These data suggested that colonic mucosal immunity was somewhat improved during cold exposure when dietary glucose was adequate.

## Discussion

Chronic cold stress is a frequent face challenge for pigs at high latitudes in autumn and winter. Low temperatures in the cold region promote the risk of disease in pigs, which undoubtedly increases the burden on farming and may promote antibiotic use. Mammals cope with prolonged exposure to cold by modulating their endocrine and metabolic systems, and high energy expenditure. Vast amounts of glucose and triglycerides are consumed in this process, whether the animal can adapt to low temperatures. Moreover, the gut microbiota is vital in modulating the host metabolism during cold adaptation [19]. Under cold stress, mammals show active resistance, adaptation, and irreversible detrimental effects [26]. Our previous findings have shown that Min pigs are less adversely affected during chronic cold exposure. In contrast, Yorkshire pigs have varying degrees of damage to the small intestine [6], lungs [11], and heart [27] induced by chronic cold stress. This evidence implies that chronic cold exposure significantly impacts Yorkshire pigs. Different pig breeds may have different tolerance to low temperatures. We used the Min pig to develop the cold adaptation model, which is usually recognized to have good low-temperature tolerance. The Yorkshire pig, which has weak low-temperature adaptability, was chosen as the non-cold adaptation model. This study revealed the differential response of adaptive and non-adaptive glucose and lipid metabolic programs during chronic cold exposure. Furthermore, we also characterized differences in gut microbiota-mediated colonic mucosal immunity during cold and non-cold adaption. Moreover, the effects of glucose as an energy supplement on regulating glucose and lipid metabolism and colonic mucosal immunity in cold-exposed pigs were evaluated.

Certainly, cold intensifies energy-consuming processes. It has been shown that cold exposure enhances thermogenesis by promoting glucose breakdown [14]. First, we found that cold exposure decreased plasma glucose levels in Yorkshire pig models, which was not observed in Min pig models. Glucose is highly hydrophilic and cannot move freely through hydrophobic biofilms, requiring the assistance of transmembrane transporters to enter cells [15]. Free glucose is absorbed by GLUT2 and SGLT1 transport in the duodenum and jejunum [28]. The *SGLT1* mRNA expression in the duodenum of the MCS group was increased significantly during cold exposure. This indicated that the Min pigs' jejunal mucosa might have a stronger ability to absorb exogenous glucose, providing more energy for the body. However, glucose transport in the jejunal mucosa of Yorkshire pigs was unaffected. The liver is one of the primary sites of glucose metabolism and the core of energy metabolism. Glycolysis converts glucose to pyruvate by hexokinases (HK) and pyruvate kinases (PK) [29–31]. Pyruvate enters the tricarboxylic acid cycle and ultimately releases ATP. When glucose is consumed in large quantities, the glycogenolysis and gluconeogenesis pathways produce glucose to meet demand. Glycogen decomposition depends on PYGL [32]. Gluconeogenesis is regulated by PEPCK, G6PC and PC [33–35]. Here, higher PK, PC and G6PC concentrations were detected in the liver of the MCS group than in the MC group. Both glycolytic and gluconeogenic pathways were enhanced in Min pigs. We found a similar phenomenon in Yorkshire pigs. The PK and G6PC concentrations were higher in the YCS group than in the YC group. Nevertheless, it is intriguing to note that cold exposure increased PYGL activity in the livers of Min pigs. Glucagon will likely cause hepatic glycogenolysis [36, 37]. Normally, glucagon activates the glucose-producing pathway (gluconeogenesis and glycogenolysis) in the liver when the body's plasma glucose is at low levels [38, 39]. The glucagon-mediated glycogenolysis pathway was activated in Min pigs, which ensured glucose supply and maintained plasma glucose homeostasis during cold exposure. Glucagon, the glucose counter-regulatory hormone, raises plasma glucose by boosting hepatic gluconeogenesis and glycogenolysis. This might be an important mechanism for maintaining glucose homeostasis in Min pig models during cold exposure. Moreover, the characterization of the glycolysis in the muscle was different between the two models. The muscle of Yorkshire pigs exposed to the cold had more active glycolysis with the GLUT1 and *HK2* upregulation. GLUT1 is among the essential glucose transporters in the pig muscle [40], responsible for glucose exchange between the muscle and blood. *HK2* is among the main isozymes responsible for the first step in glycolysis [41]. According to our findings, Yorkshire pigs ingested more glucose in the muscle during cold weather. Although we could not conclusively determine whether the drop in plasma glucose in the Yorkshire pig models during cold exposure was caused by an inactive glucagon pathway, we must concede that Yorkshire pigs overconsumed glucose.

Triglycerides and glucose are significant fuels for active cold adaptation in animals, both for non-shivering thermogenesis in brown adipose tissue and for ATP supply to cells [42, 43]. Ketone bodies are widely regarded as one of the products of lipolysis. They are produced when insufficient glucose is available in the liver [44]. KEGG analysis of plasma metabolomics revealed that ketosome synthesis and degradation pathways were highly active in Yorkshire pigs during chronic cold exposure. Recent research reveals that ATGL, the primary, lipolytic enzyme, is necessary for mammals' cold adaptation [13]. ATGL is the rate-limiting enzyme that breaks down fat into large amounts of fatty acids [45, 46]. After triglyceride is hydrolyzed to fatty acid, abundant ATP is produced by fatty acid oxidation to meet cellular energy demands. Since the CPT-1α-catalyzed acyl transfer to carnitine is among the rate-limiting steps during fatty acid oxidation, CPT-1α enriched in the liver is indispensable for fatty acid oxidation [47, 48]. *PPARα* can enhance CPT-1α to promote the β-oxidation of fatty acids, thereby increasing energy expenditure [49, 50]. Our data illustrated that the expression of ATGL, CPT-1α and *PPARα* expression is up-regulated during cold exposure in Yorkshire pig models with glucose overconsumption. Thus, both lipolysis and fatty acid oxidation were increased in Yorkshire pigs. However, Min pig models did not react to the cold. It may be established that the increase in lipolysis and fatty acid oxidation during cold exposure was caused by the increased ingestion of glucose. By increasing the glucose levels in the diet and using Yorkshire pigs as models for cold exposure, we attempted to provide evidence for this theory. The *CD36*, *FATP1*, *PPARα*, *ATGL* and *CPT-1α* expression was inhibited in the liver when the dietary glucose supply was adequate. Lipolysis and fatty acid oxidation remain inactive when glucose sufficiently covers the body's heat demands. Thus, cold-induced glucose overconsumption promotes lipolysis and fatty acid oxidation in the liver to maintain normal body temperature. Moreover, during chronic cold exposure, the *CD36* and *ACC* expression in the longissimus dorsi muscle were inhibited in Yorkshire pigs. The *CPT-1a* expression in the dorsal fat was enhanced. *ACC* is involved in the metabolic pathway for de novo fatty acid synthesis [51]. CD36 is important in fatty acid transport or lipid utilization [52, 53]. Chronic cold exposure promoted fatty acid oxidation in the dorsal fat and inhibited fat synthesis and lipid transport in the muscle of Yorkshire pigs. Intriguingly, LXR was activated in the liver of Min pigs during cold exposure. *LXRα* is one of the key genes in adipose synthesis [54, 55]. The fatty acid transporter *CD36* in the liver is an important target gene for LXR [56]. Fatty acid transport protein 1 (FATP1) is a fatty acid transport protein involved in fatty acid uptake [57]. Interestingly, previous evidence has shown that LXR inhibits proinflammatory cytokines such as IL-6 [58]. LXR-mediated anti-inflammatory and fat metabolism might have contributed to the better cold tolerance of pigs, but the additional proof was required. We attempted to improve plasma glucose in Yorkshire pigs during cold exposure to dietary glucose supplements. Regarding evaluating glucose transport ability in the jejunal mucosa, *SGLT1* mRNA expression was higher in the G group than in the C group, indicating that the glucose absorption capacity of the small intestine of cold-exposed pigs was enhanced. Moreover, the data showed that dietary glucose supplements elevated plasma glucose levels and blocked the glucogenic pathway (gluconeogenesis and glycogenolysis) and lipolysis. Thus, pigs did not need to mobilize their energy reserves to combat the cold when energy intake was adequate.

The metabolism of the microbiome affects the host's energy metabolism, and the gut microbiota influences host immunity in the intestinal tract and at distal sites [59]. In this research, cold exposure generated substantial changes in the microbiota composition, reducing the ratio of Firmicutes to Bacteroidetes. It is commonly accepted that a high Firmicutes/Bacteroidetes ratio (decrease in Bacteroidetes, increase in Firmicutes) has been associated with fat deposition [60, 61]. Cold decreased Firmicutes/Bacteroidetes ratio in both pig models during cold exposure. The microbiota's preference for energy deposition was reduced. However, significant differences remain between the gut microbiota responses of the two models during cold exposure. *Collinsella* is thought to benefit host metabolism and immunity [62]. *Bifidobacterium* is a widely recognized probiotic that produces butyric acid [63]. Interestingly, a restraint in the abundance of probiotics was detected in the colon microbiota of Yorkshire pigs, such as *Collinsella* and *Bifidobacterium* depletion in Actinobacteria. A recent study has shown that *Sutterella* does not appear to cause substantial colonic inflammation but can degrade IgA [64]. There is no doubt that *Escherichia-Shigella* is widely recognized as a typical pathogenic bacterium [65–67]. Notably, the prevalence of *Sutterella* and *Escherichia-Shigella*, which were widely thought to be pathogenic bacteria, was more enriched. These microbiota alterations were harmful to colonic mucosal immunity. Additionally, we found that *Prevotella*, *Prevotellaceae_NK3B31_group* and *Prevotellaceae_UCG_004* abundance was enriched, while *Coprococcus* was diminished in the YCS group. *[Ruminococcus]_torques_group* and *[Eubacterium]_coprostanoligenes_group* abundance was reduced in the YCS group. *Prevotella*, *Prevotellaceae_NK3B31_group* and *Prevotellaceae_UCG_004* are important members of *Prevotellaceae*. *Prevotellaceae* helps to aggravate colitis [68]. Although some studies have shown that *Prevotellaceae_NK3B31_group* can produce short-chain fatty acids (SCFAs) [69, 70], we have not observed any changes in SCFAs between the YC and YCS groups. This was probably related to the depletion of *Collinsella* and *Bifidobacterium.* Meanwhile, we also observed the inflammatory infiltration and nuclear structure injury in the colon of Yorkshire pig models, accompanied by the activation of the inflammatory pathway and the damage of the mechanical barrier. Tight junction (TJ) structural proteins, such as ZO-1, Occludin, and Claudin, establish the mechanical intestinal barrier [71]. The Toll-like receptors 4 (TLR4) pathway is involved in inflammatory responses in many diseases, and the lipopolysaccharide (LPS) is an exogenous ligand of TLR4 [72, 73]. It has been reported that the Myd88-dependent pathway is mediated by TLR4/Myd88/NF-κB activation and proinflammatory factor production (such as IL-1β, IL-6 and TNF-α) [74]. Moreover, TLR4 regulates the Bcl-2 and Bax (key roles in apoptosis) expression [75]. Here, higher expression of *IL-1β*, *IL-6*, *IL-2*, TLR4, MyD88, Caspase1 and Bax was observed in the colonic mucosa of the YCS group. These results confirmed that cold exposure altered Yorkshire pigs' gut microbiota, disrupted the colon mucosal barrier, and induced inflammation and apoptosis. In addition to producing ATP, mitochondria produce a great deal of ROS. ROS generation is largely driven by mitochondrial malfunction [76]. The damaged mitochondria are cleaned up by mitochondrial autophagy (mitophagy), an organelle quality control pathway [77, 78]. Mitochondrial dynamics are regulated by fission and fusion. Mitofusin 1 (Mfn1), mitofusin 2 (Mfn2), and optic atrophy 1 (Opa1) are involved in regulating mitochondrial fusion, respectively [42]. BNIP3 can stimulate mitochondrial network fragmentation, and promote the phagocytosis of damaged mitochondria and apoptosis [79, 80]. We found that the BNIP3, *Mfn1*, *Mfn2*, and *OAP1* expression in the colonic mucosa of the YCS group was increased by cold exposure, suggesting that chronic cold stress promoted mitochondrial autophagy and mitochondrial fusion in the colonic mucosa of the YCS group. In a word, cold exposure disrupted the colon microbiota and impaired the colon mucosal barrier and function. We also examined the regulatory effects of dietary glucose supplements on colonic mucosal immunity and function in cold-exposed pigs. During cold exposure, glucose supplementation lowered the *IL-1β* and *IL-6* expression in the colonic mucosa of the cold-exposed pigs more than in the C group. Moreover, glucose supplementation inhibited apoptosis, accompanied by the down-regulation of Bax and up-regulation of Bcl2. This evidence suggested that supplementing Yorkshire pigs with glucose during cold exposure might be advantageous.

In contrast, the mucosal immunity and microbiota of the colon were less disturbed in Min pigs. The abundance changes of *Rikenellaceae_RC9_gut_group*, *[Eubacterium] _coprostanoligenes_group*, *UCG-010*, *Bacteroidales_RF16_group* and *WCHB1-41* contributed to the metabolic adaptation and might protect the colonic epithelial barrier during the cold. Many studies have shown that *Rikenellaceae_RC9_gut_group* abundance negatively correlates with blood glucose [81]. Its enrichment contributed to cold-adapted blood glucose homeostasis during cold exposure. *[Eubacterium]_coprostanoligenes_group* are beneficial metabolization-related bacteria that can convert cholesterol to coprostanol [82]. *Rikenellaceae_RC9_gut_group* benefits the gut homeostasis, potentially SCFA-producing bacteria [83, 84]. Recent studies have shown that *WCHB1-41* significantly regulates nutritional requirements during cold [85]. These changes in the genus levels were beneficial to the cold adaptation of the Min pig models during cold exposure, both immunologically and metabolically. We found that the mRNA expression of *pBD-2* and *PR-39* was enhanced in the colonic mucosa of Min pigs during chronic cold exposure. They are key members of the colonic mucosa's defense peptides [22], which prevent the invasion of pathogenic microorganisms. Straight-chain and branched volatile fatty acids produced by the colonic microbiota contribute to colonic immunity [86]. Recently, several investigations revealed the benefit of valeric acid and isovaleric acid, although they only occupy a small proportion of the total shorter-chain fatty acids in the colon [87, 88]. During cold exposure, the colon contents of Min pigs showed astonishingly high quantities of valeric acid and isovaleric acid during cold. However, we did not directly demonstrate that colonic microbiota and volatile fatty acids were essential in factors for protecting the Min pig models from the negative impacts of cold exposure. Our data affirmingly confirmed that the colonic mucosal barrier in the Min pigs was not negatively affected by the cold.

## Conclusion

**Fig. 9:**
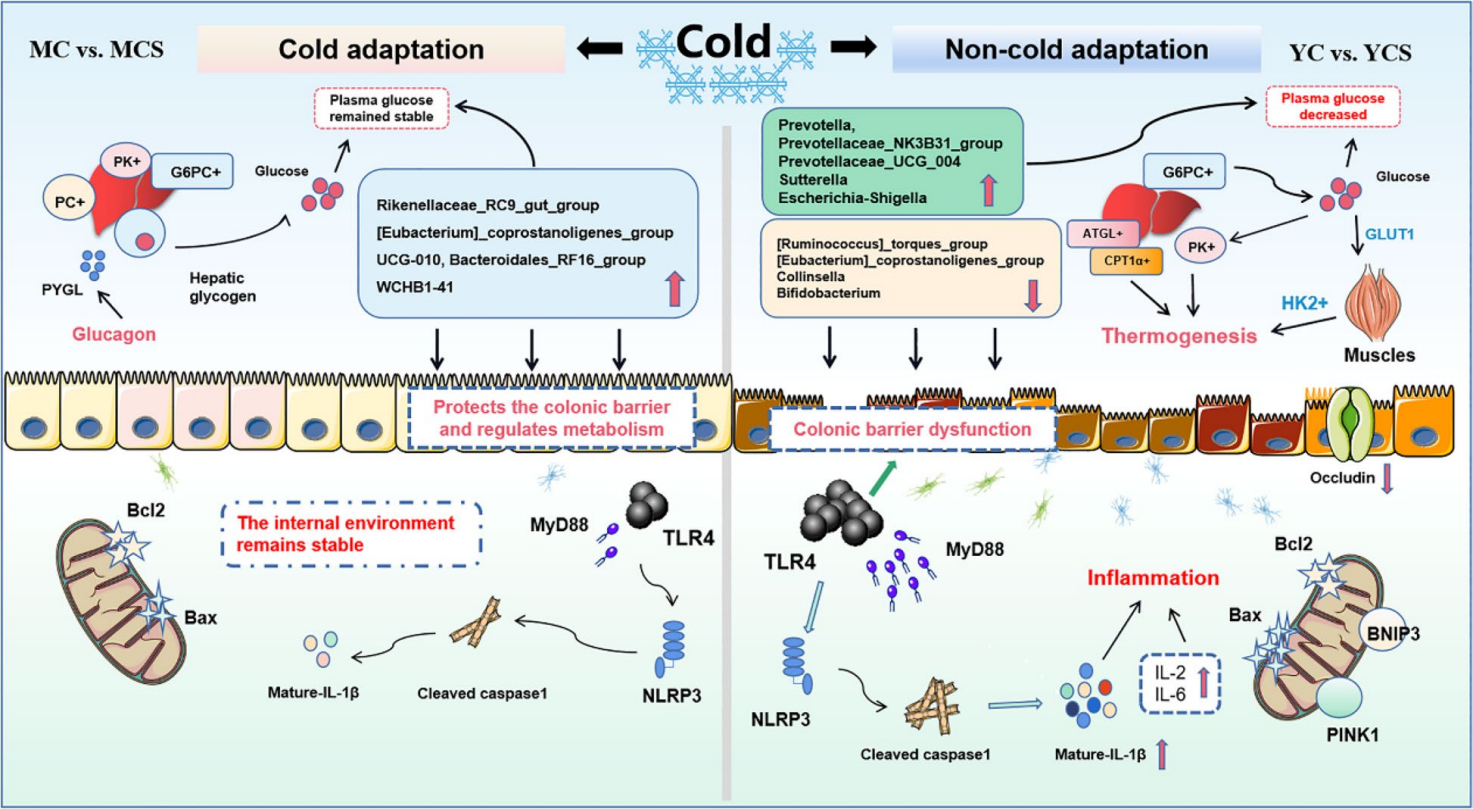
Cold-induced glucose overconsumption drives lipolysis but interferes with the gut microbiota. We delineate the importance of glucagon-mediated hepatic glycogenolysis for glucose homeostasis during cold exposure by using Min pig (cold-adapted) models and Yorkshire pig (non-cold-adapted) models. Moreover, we reveal that lipolysis and fatty acid oxidation under chronic cold exposure result from excessive glucose consumption. It should be particularly emphasized that cold-induced glucose overconsumption is detrimental to colonic microbiota and mucosal immunity, although lipolysis and fatty acid oxidation contribute to thermogenesis to maintain a stable internal body temperature

In summary, using Min pig (cold-adapted) models and Yorkshire pig (non-cold-adapted) models, we delineate the importance of glucagon-mediated hepatic glycogenolysis for glucose homeostasis during cold exposure. Moreover, we found that lipolysis and fatty acid oxidation were promoted during cold exposure when excessive glucose was consumed. It should be underlined that cold exposure is deleterious to colonic microbiota and mucosal immunity, despite the contribution of lipolysis and fatty acid oxidation to thermogenesis, which helps maintain a steady internal body temperature (Fig. 9). Dietary glucose can regulate glucose and lipid metabolism and colonic mucosal immunity in cold-exposed pigs. Our findings provide new evidence for the characteristics of glucose and lipid metabolism and the interaction between microbiota and colonic mucosa in cold and non-cold adapted pigs, and a new nutrition strategy is proposed for cold exposed pigs.

## Supplementary Information

**Additional file 1:** **Table S1**. Composition of experimental diets for Min pigs and Yorkshire pigs.

**Additional file 2:** **Table S2**. Composition of high glucose diets.

**Additional file 3:** **Table S3**. Details of primers involved in qPCR.

**Additional file 4:** **Table S4**. Details of primary and secondary antibodies involved in Western blot.

**Additional file 5:** **Table S5**. Differential metabolites enriched in the plasma metabolome.

**Additional file 6:** **Table S6**. Differential metabolites enriched in the plasma metabolome.

**Additional file 7:** **Fig. S1.** Regulation of hormones and plasma metabolites in Min pigs and Yorkshire pigs by cold exposure.

**Additional file 8:** **Fig. S2.** Effects of chronic cold exposure on glucose transporters in jejunal mucosa of Min pigs and Yorkshire pigs.

**Additional file 9:** **Fig. S3.** Glucose metabolism responses in the liver and peripheral tissue of Min and Yorkshire pigs during cold exposure.

**Additional file 10:** **Fig. S4.** Short chain fatty acidsin the colonic content of Min pigs and Yorkshire pigs during cold exposure.

**Additional file 11:** **Fig. S5.** Effects of cold exposure on colonic mucosal function of Min and Yorkshire pigs.

**Additional file 12:** **Fig. S6.** Effects of chronic cold exposure on genes related to lipid metabolism in the dorsal fat and longissimus dorsi muscle of Min pigs and Yorkshire pigs.

**Additional file 13:** **Fig. S7.** Effects of dietary glucose supplementation on glucose transporters in jejunal mucosa of cold-exposed Yorkshire pigs.

## Data Availability

The datasets produced and/or analyzed during the current study are available from the corresponding author on reasonable request. The raw data of the 16S rDNA gene sequencing has been shared in the NCBI databases (https://www.ncbi.nlm.nih.gov/sra/PRJNA886839).

## Abbreviations

ATGL: Adipose TG lipase
Bax: Bcl-2- associated X-protein
Bcl-2: B-cell lymphoma 2
BNIP3: Bcl-2/adenovirus E1B 19-kDa interacting protein
CD36: Fatty acid transport protein cluster of differentiation 36
C/EBPα: Transcription factor CCAAT enhancer binding protein α
CPT-1α: Carnitine palmitoyl transferase 1α
FAS: Fatty acid synthase
FATP1: Fatty acid transporter 1
G6PC: Glucose-6-phosphate kinase
GLUT1: Glucose transporter 1
GLUT2: Glucose transporter 2
GLUT4: Glucose transporter 4
GS: Glycogen synthase
HK: Hexokinase
HK2: Hexokinase type 2
LXRα: Nuclear receptor liver X receptor alpha
Mfn1: Mitofusin 1
Mfn2: Mitofusin 2
MyD88: Myeloid differentiation main response 88
NLRP3: Nucleotide-binding domain and leucine-rich repeat protein 3
OPA1: Mitochondrial dynamin like GTPase
pBD-1: Porcine β-defensin 1
pBD-2: Porcine β-defensin 2
PC: Pyruvate carboxylase
PEPCK: Phosphoenolpyruvate kinase
PFKM: Phosphofructokinase, muscle
PINK1: PTEN induced kinase 1
PK: Pyruvate kinase
PKM: Pyruvate kinase M
PPARα: Peroxisome-proliferator-activated receptor alpha
PYGL: Glycogen phosphorylase
SGLT1: Sodium-glucose cotransporter 1
TLR4: Toll-like receptor 4
T3: Triiodothyronine
T4: Thyroxine
