# Supplementary material for: Characteristics of glucose and lipid metabolism and the interaction between gut microbiota and colonic mucosal immunity in pigs during cold exposure
Source: J Anim Sci Biotechnol. 2023 Jul 4;14:84. doi: 10.1186/s40104-023-00886-5 (PMC10318708; doi:10.1186/s40104-023-00886-5)
Supplement: Supplementary file 1 — Additional file 1: Table S1. Composition of experimental diets for Min pigs and Yorkshire pigs. [file 40104_2023_886_MOESM1_ESM.docx]

**Table S1** Composition of experimental diets

| **Basic diet ingredients** | | **Content, %** |
| --- | --- | --- |
|  | Corn | 73.00 |
|  | Soybean meal, de-hulled | 15.30 |
|  | Full-fat soybean meal, puffed | 5.00 |
|  | Fish meal | 2.00 |
|  | Soybean oil | 1.00 |
|  | *L*-Lysine | 0.39 |
|  | *DL*-Methionine | 0.04 |
|  | *L*-Threonine | 0.12 |
|  | *L*-Tryptophan | 0.02 |
|  | Calcium hydrogen phosphate | 1.19 |
|  | Limestone | 0.66 |
|  | Salt | 0.28 |
|  | Premix^a^ | 1.00 |
| Nutrient levels^b^ | |  |
|  | NE, Mcal/kg | 2.50 |
|  | Crude protein | 16.03 |
|  | Lysine | 0.98 |
|  | Methionine | 0.29 |
|  | Threonine | 0.60 |
|  | Leucine | 0.17 |
|  | Calcium | 0.66 |
|  | Total phosphorus | 0.56 |
|  | Available phosphorus | 0.33 |
|  | Sodium | 0.14 |
|  | Chlorine | 0.19 |

^a^Provided the following per kilogram of diet: Fe, 160 mg; Cu, 150 mg; Mn, 40 mg; Zn, 140 mg; Se, 0.4 mg; I, 0.5 mg; vitamin A, 8,000 IU; vitamin D_3_, 2,000 IU; vitamin E, 30 mg; vitamin B_1_, 1.60 mg; vitamin B_2_, 5.00 mg; vitamin B_6_, 5.00 mg; vitamin B_12_, 0.01 mg; pantothenic acid, 20 mg; niacin, 15 mg; biotin, 0.05 mg

^b^Nutrient levels were calculated values
