# Supplementary material for: Characteristics of glucose and lipid metabolism and the interaction between gut microbiota and colonic mucosal immunity in pigs during cold exposure
Source: J Anim Sci Biotechnol. 2023 Jul 4;14:84. doi: 10.1186/s40104-023-00886-5 (PMC10318708; doi:10.1186/s40104-023-00886-5)
Supplement: Supplementary file 3 — Additional file 3: Table S3. Details of primers involved in qPCR. [file 40104_2023_886_MOESM3_ESM.docx]

**Table S3** The real-time PCR primers

| **Gene** | **GenBank ID** | **Primer sequences (5' to 3')** |
| --- | --- | --- |
| *β-actin* | AY550069 | F: ATGCTTCTAGGCGGACTGT |
|  |  | R: CCATCCAACCGACTGCT |
| *GLUT1* | XM_021096908.1 | F: ATCATCGGTGTGTACTGCGG |
|  |  | R: TCGTTGCCCATGATGGAGTC |
| *GLUT2* | NM_001097417.1 | F: GACACGTTTTGGGTGTTCCG |
|  |  | R: GAGGCTAGCAGATGCCGTAG |
| *GLUT4* | AB005285.1 | F: TTTGACCAGATCTCAGCCGTC |
|  |  | R: GTCATTCTCATCCGGCCCTA |
| *SGLT1* | NM_001164021.1 | F: GCAACAGCAAAGAGGAGCGTAT |
|  |  | R: GCCACAAAACAGGTCATAGGTC |
| *HK2* | NM_001122987.1 | F: CAGGAGATCGACATGGGCTC |
|  |  | R: GTTCAGGACTGAGCTTCCCC |
| *PKM* | XM_021099125.1 | F: GGCCCTGCAGTGGAGATAAC |
|  |  | R: TTGTTGGTTCAGGGTGGCTC |
| *PFKM* | NM_001044550.1 | F: GGAGAGCTGAGACTATAAGAGTGG |
|  |  | R: GCAGCATTCATTCCTTGGGC |
| *Bax* | XM_005664710 | F: ATGGAGCTGCAGAGGATGAT |
|  |  | R: AAAGTAGAAAAGCGCGACCA |
| *Bcl2* | NM_001164511.2 | F: ACTTCTGCGAAAGCGAATTGCC |
|  |  | R: AGCCTCCGTTTTGCCTTATCC |
| *Caspase3* | NM_214131 | F: CGGACAGTGGGACTGAAGTA |
|  |  | R: GATCCGTCCTTTGAATTTCG |
| *NLRP3* | NM_001256770.2 | F: CTGGGACTCTGACTAGGGCT |
|  |  | R: TTTTTCTGTCTGGCCCCGAG |
| *HMGB1* | NM_001004034.1 | F: GAGGAAACTTGAGACCCACCA |
|  |  | R: GTGTCCTTCCTTCCCTCATGT |
| *Caspase1* | NM_214162.1 | F: TACAAGAATCCCAGGCGGTG |
|  |  | R: CCTTTGGGCTATGTCTGGGG |
| *TLR4* | NM_001113039.2 | F: CAGTCAAGATACTGGACCTGAGC |
|  |  | R: GGCTCCCAGGGCTAAAACTCT |
| *MyD88* | NM_001099923.1 | F: CCATTCGAGATGACCCCCTG |
|  |  | R: TAGCAATGGACCAGACGCAG |
| *IL-1β* | NM_2140551.1 | F: GCCAACGTGCAGTCTATGGAGTG |
|  |  | R: GGTGGAGAGCCTTCAGCATGTG |
| *PC* | NM_214349.1 | F: GTGCTCTCAGCAGCCATGTA |
|  |  | R: GTCACTTATGGCCAGGGCTT |
| *PCK2* | XM_021098318.1 | F: TGAGCTCCCTGAGAAAGGTAGA |
|  |  | R: CTGCCTCAGTCCCATCACAG |
| *G6PC* | EU717834.1 | F: AACCCCCATCCCAAATCGAG |
|  |  | R: AGGACGCCACATCTGTCTAC |
| *FAS* | NM_213839 | F: CACACCAACCAGCAACACCAAATG |
|  |  | R: AGGTACGGGAATGAGGATCAGGAG |
| *LXRα* | NM_001101814.1 | F: ACGCTTTGCCCACTTCACTGAG |
|  |  | R: GTCTCCAGAAGCATCACCTCAATCG |
| *CD36* | NM_001044622 | F: CGTACAGAGTTCGTTATCTAGCCAAGG |
|  |  | R: AGCCAGATTGAGAACAGTGAAGGTG |
| *FATP1* | NM_001004046 | F: GGACATCAAGGGGACATCGGAAATC |
|  |  | R: GGTCTCCATCTCACACTCCTCTCC |
| *PPARα* | NM_001044526.1 | F: TGGCTTACGGCAATGGCTTCATC |
|  |  | R: GCCACAAAGAGGGAAAGGTCACTG |
| *APOB* | NM_001375388.1 | F: AAGCCAGAGCCTACCTCCACATC |
|  |  | R: ACGAACACCATTCAGCAGCAGTC |
| *ATGL* | EU373817.1 | F: ATGGTGCCCTACACGCTG |
|  |  | R: GCCTGTCTGCTCCTTTATCC |
| *CPT-1α* | NM_001129805.1 | F: ACTGTCTGGGCAAACCAAAC |
|  |  | R: CTTCTTGATGAGGCCTTTGC |
| *ACC* | AF175308.1 | F: CCGGAATATCCAGAAGGCCG |
|  |  | R: CCAGTCCGATTCTTGCTCCA |
| *C/EBPα* | XM_003127015.4 | F: CTCACCGCTCCGATTCCTAC |
|  |  | R: TCCTTCTATTGCGGGGGAGA |
| *IL-2* | NM_213861 | F: AGCTCTGGAGGGAGTGCTAA |
|  |  | R: ACAGCAGTTACTGTCTCATCATCA |
| *IL-6* | NM_214399 | F: ACAAAGCCACCACCCCTAAC |
|  |  | R: CGTGGACGGCATCAATCTCA |
| *IFN-γ* | NM_214304.1 | F: ATGAGGCAGAGTACCGGGAT |
|  |  | R: AGCGTAAGCATTGCGGATCT |
| *PR-39* | L23825.1 | F: GAACCCATCCATTCACTCAC |
|  |  | R: TTATCAGCCACTCCATCACC |
| *pBD-1* | NM_213838.1 | F: CCAGAGGTCCGACCACTACA |
|  |  | R: GGTCCCTTCAATCCTGTTGAA |
| *pBD-2* | AY506573.1 | F: CCAGAGGTCCGACCACTACA |
|  |  | R: GGTCCCTTCAATCCTGTTGAA |
| *Mfn1* | NM_001315732.1 | F: TGGACTTTATCCGAAACCAGATGAACC |
|  |  | R: AACCTTATTTGCCACCTCCTCTGTAAC |
| *Mfn2* | XM_021095369.1 | F: CCACACCACCAACTGCTTCCTG |
|  |  | R: TCTTGACGCTCCTCTTCTCCTCTG |
| *OPA1* | XM_021070063.1 | F: ACAGAGGATGGTGCTTGTTGACTTAC |
|  |  | R: ACACAGTATGATGGCGTTGGGATTC |
| *Fis1* | XM_021086263.1 | F: CAGACAGAGCCACAGAACAACCAG |
|  |  | R: CAAGTCCAATGAGTCCAGCCAGTC |
| *MFF* | NM_001244126.1 | F: CAGGTTCCAGAGAGAATTGTCGTAGC |
|  |  | R: TTAGTGCCAGAGGTTTAAAGGGAGTTG |
| *BNIP3* | XM_003359404.4 | F: GAGGAGGATTACATGGAGAGGAGGAG |
|  |  | R: TCGGGTGCTTGAAGAGGAGGAAC |
| *PINK1* | XM_021095478.1 | F: GGCGGTGATTGACTACAGCAAGG |
|  |  | R: TGGTAACTGCGGCTTTCAAGGTG |
| *p62* | NM_001244307.1 | F: CTGCCTGAAGACTATTACACGAGACC |
|  |  | R: GAAGATGCTTGTGCCGAGGATAGAG |
| *LC3I* | NM_001170827.1 | F: GCCTTCTTCCTGCTGGTGAACC |
|  |  | R: GGGAGGCGTAGACCATGTAGAGG |
| *LC3II* | NM_001190290.1 | F: TTCTTCCTGTTAGTGAACGGACATAGC |
|  |  | R: ATCCATCTTCATCCTTCTCGCTTTCG |
| *ZO-1* | XM_005659811.1 | F: AGCCATCCACTCCTGCCTAT |
|  |  | R: GGGACCTGCTCATAACTTCG |
| *Occludin* | NM_001163647.2 | F: GAGTACATGGCTGCTGCTGA |
|  |  | R: AACAAGGTGGCCTCTGTCTC |
| *Claudin-1* | NM_001244539.1 | F: GCCCTACTTTGCTGCTCCTG |
|  |  | R: TTTCTGGTTGTTCCCACACG |
