# Supplementary material for: Characteristics of glucose and lipid metabolism and the interaction between gut microbiota and colonic mucosal immunity in pigs during cold exposure
Source: J Anim Sci Biotechnol. 2023 Jul 4;14:84. doi: 10.1186/s40104-023-00886-5 (PMC10318708; doi:10.1186/s40104-023-00886-5)
Supplement: Supplementary file 7 — Additional file 7: Fig. S1. Regulation of hormones and plasma metabolites in Min pigs and Yorkshire pigs by cold exposure. [file 40104_2023_886_MOESM7_ESM.docx]

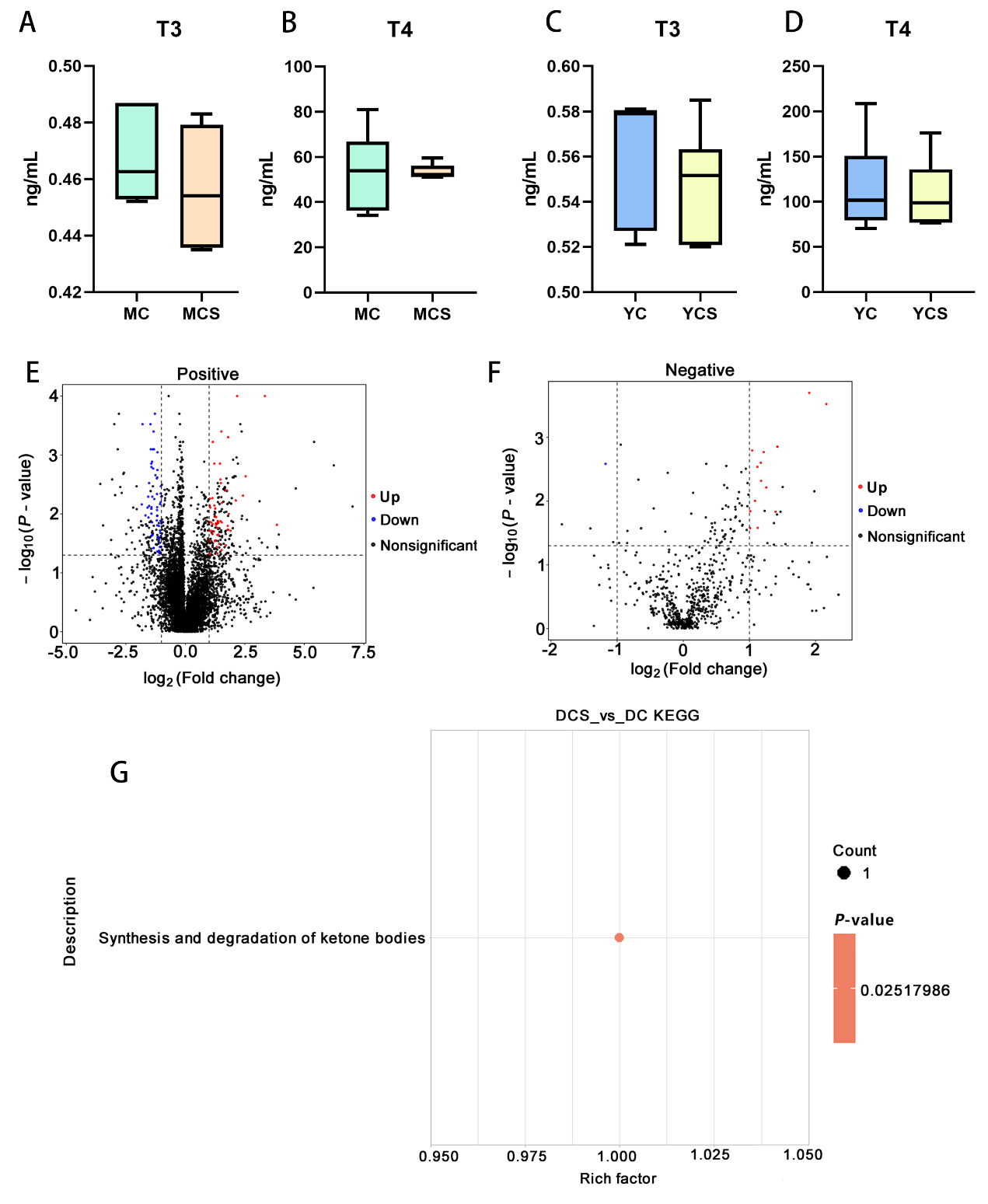


**Fig. S1** Regulation of hormones and plasma metabolites in Min pigs and Yorkshire pigs by cold exposure. **A** Phenotypes of Min pigs. **B** Phenotypes of Yorkshire pigs. **C** and **D** Plasma hormones of Min pigs. *n* = 6. ^*^*P* < 0.05. **E** and **F** Plasma hormones of Yorkshire pigs. ^*^*P* < 0.05. **G** and **H** Volcanic map of differential metabolites in plasma of Yorkshire pigs. **I** KEGG analysis of differential metabolites in plasma of Yorkshire pigs
