# Supplementary material for: Characteristics of glucose and lipid metabolism and the interaction between gut microbiota and colonic mucosal immunity in pigs during cold exposure
Source: J Anim Sci Biotechnol. 2023 Jul 4;14:84. doi: 10.1186/s40104-023-00886-5 (PMC10318708; doi:10.1186/s40104-023-00886-5)
Supplement: Supplementary file 9 — Additional file 9: Fig. S3. Glucose metabolism responses in the liver and peripheral tissue of Min and Yorkshire pigs during cold exposure. [file 40104_2023_886_MOESM9_ESM.docx]

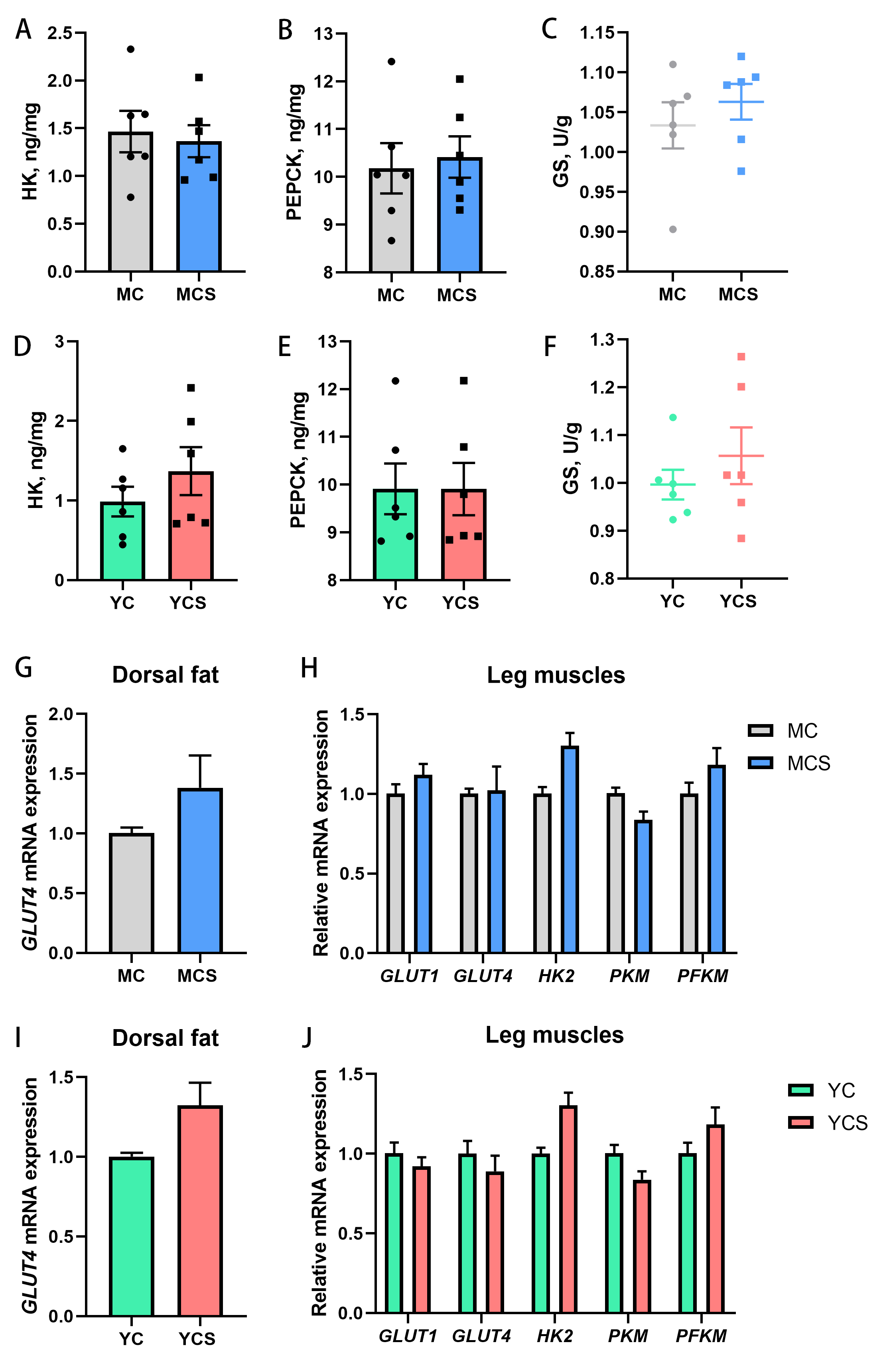


**Fig. S3** Glucose metabolism responses in the liver and peripheral tissue of Min and Yorkshire pigs during cold exposure. **A** and **B** Concentration of glucose-metabolizing enzymes in the liver of Min pigs. *n* = 6. **C** Glycogen synthase activity in livers of Min pigs. *n* = 6. **D** and **E** Concentration of glucose-metabolizing enzymes in the liver of Yorkshire pigs. **F** Glycogen synthase activity in the liver of Yorkshire pigs. *n* = 6. **G** *GLUT4* mRNA expression in the dorsal fat of Min pigs. *n* = 6. **H** Glucose transport and glycolysis in the leg muscle of Min pigs. **I** *GLUT4* mRNA expression in the dorsal fat of Yorkshire pigs. *n* = 6. **J** Glucose transport and glycolysis in the leg muscle of Yorkshire pigs, *n* = 6. ^*^*P* < 0.05
