# Supplementary material for: Characteristics of glucose and lipid metabolism and the interaction between gut microbiota and colonic mucosal immunity in pigs during cold exposure
Source: J Anim Sci Biotechnol. 2023 Jul 4;14:84. doi: 10.1186/s40104-023-00886-5 (PMC10318708; doi:10.1186/s40104-023-00886-5)
Supplement: Supplementary file 10 — Additional file 10: Fig. S4. Short chain fatty acidsin the colonic content of Min pigs and Yorkshire pigs during cold exposure. [file 40104_2023_886_MOESM10_ESM.docx]

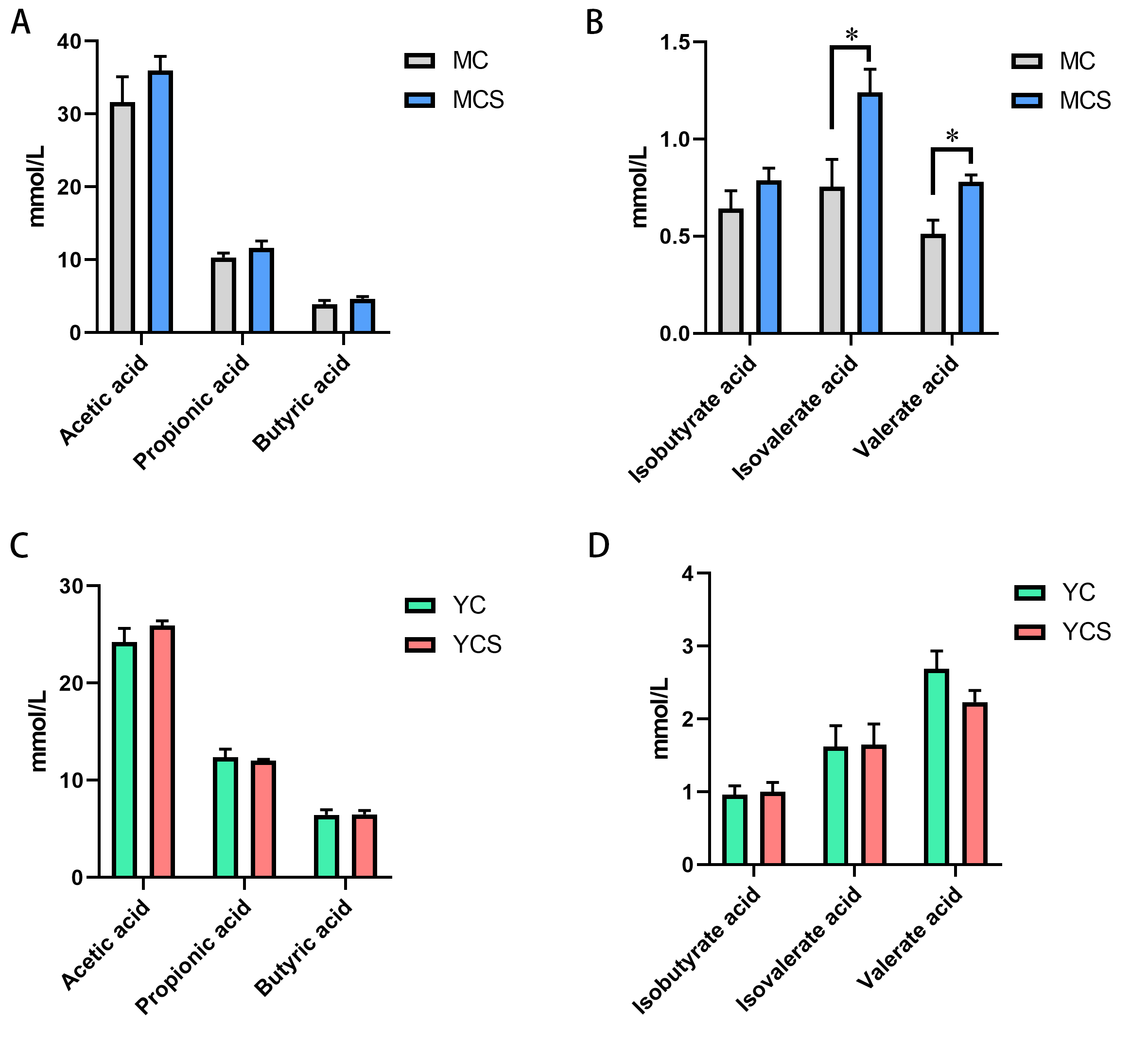


**Fig. S4** Short chain fatty acids (SCFAs) in the colonic content of Min pigs and Yorkshire pigs during cold exposure. **A** and **B** SCFAs in the colonic content of Min pigs. *n* = 5. **C** and **D** SCFAs in the colonic content of Yorkshire pigs. *n* = 5. **P* < 0.05
