# Supplementary material for: Characteristics of glucose and lipid metabolism and the interaction between gut microbiota and colonic mucosal immunity in pigs during cold exposure
Source: J Anim Sci Biotechnol. 2023 Jul 4;14:84. doi: 10.1186/s40104-023-00886-5 (PMC10318708; doi:10.1186/s40104-023-00886-5)
Supplement: Supplementary file 11 — Additional file 11: Fig. S5. Effects of cold exposure on colonic mucosal function of Min and Yorkshire pigs. [file 40104_2023_886_MOESM11_ESM.docx]

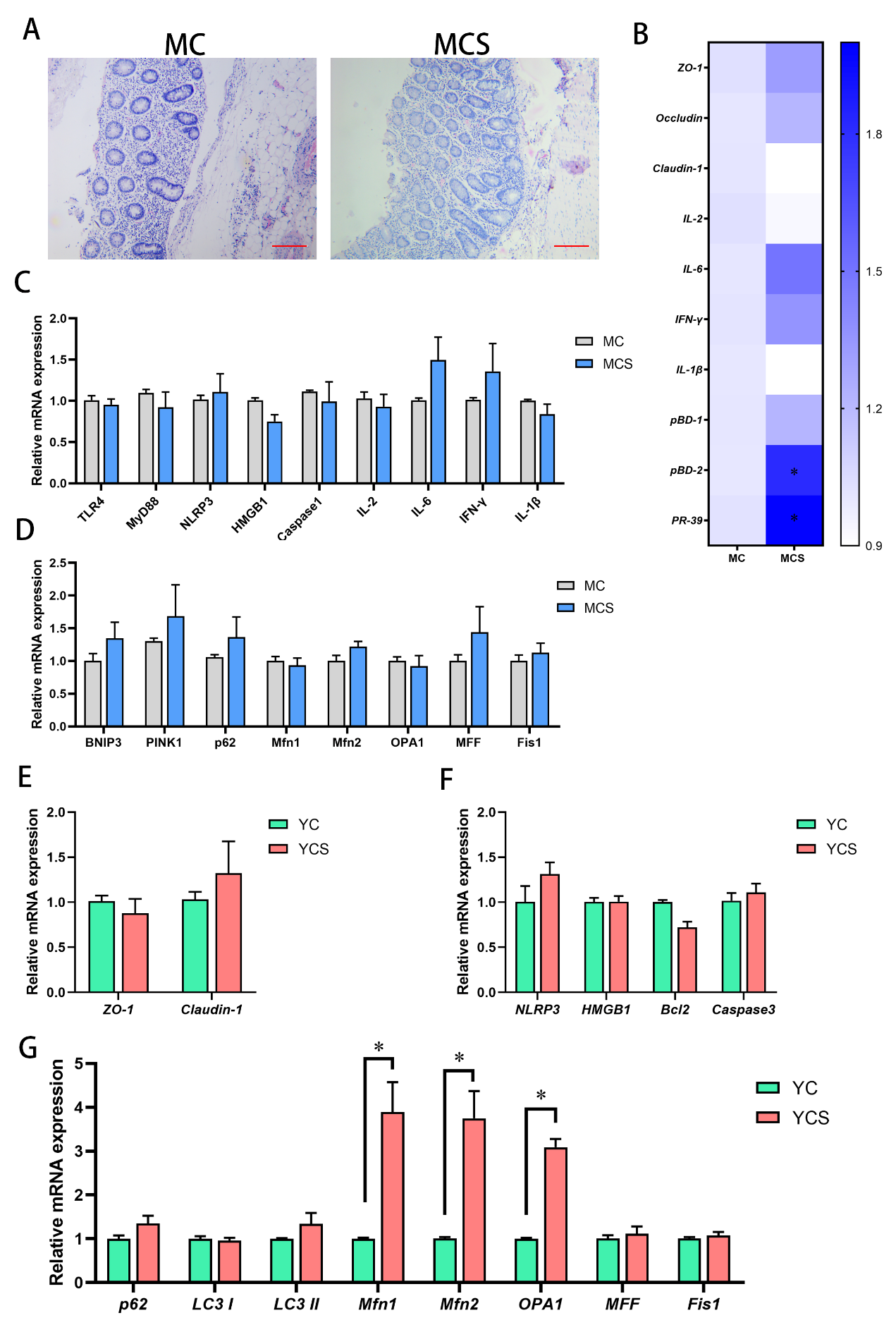


**Fig. S5** Effects of cold exposure on colonic mucosal function of Min pigs and Yorkshire pigs. **A** Pathological section of the colon in Min pigs. **B** The mRNA expression of immune factors in colonic mucosa of Min pigs. *n* = 6. **C** The mRNA expression of inflammatory pathway and apoptosis in colonic mucosa of Min pigs. *n* = 6. **D** Mitochondrial function of colonic mucosa in Min pigs. *n* = 6. **E** The mRNA expression of tight junction proteins in colonic mucosa of Yorkshire pigs. *n* = 6. **F** The mRNA expression in inflammatory pathways in the colonic mucosa of Yorkshire pigs. *n* = 6. **G** The mRNA expression for mitochondrial function in the colonic mucosa of Yorkshire pigs. *n* = 6. ^*^*P* < 0.05
