# Supplementary material for: Characteristics of glucose and lipid metabolism and the interaction between gut microbiota and colonic mucosal immunity in pigs during cold exposure
Source: J Anim Sci Biotechnol. 2023 Jul 4;14:84. doi: 10.1186/s40104-023-00886-5 (PMC10318708; doi:10.1186/s40104-023-00886-5)
Supplement: Supplementary file 12 — Additional file 12: Fig. S6. Effects of chronic cold exposure on genes related to lipid metabolism in the dorsal fat and longissimus dorsi muscle of Min pigs and Yorkshire pigs. [file 40104_2023_886_MOESM12_ESM.docx]

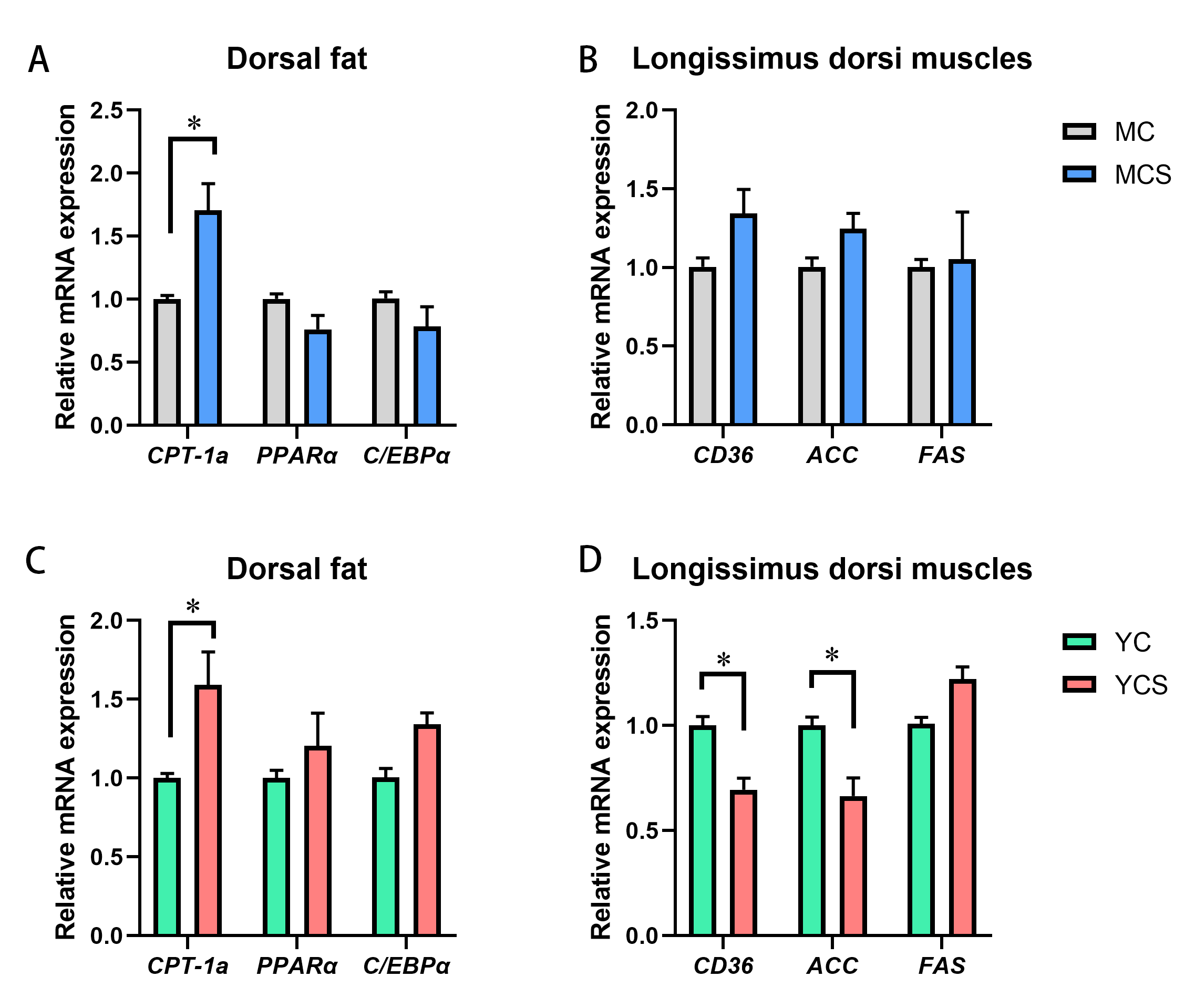


**Fig. S6** Effects of chronic cold exposure on genes related to lipid metabolism in the dorsal fat and longissimus dorsi muscle of Min pigs and Yorkshire pigs. **A** Dorsal fat of Min pigs. **B** *Longissimus dorsi* muscle of Min pigs. **C** Dorsal fat of Yorkshire pigs. **D** Longissimus dorsi muscle of Yorkshire pigs. *n* = 6. ^*^*P* < 0.05
